# Cytotoxic and Antibacterial Meroterpenoids Isolated from the Marine-Derived Fungus *Talaromyces* sp. M27416

**DOI:** 10.3390/md22040186

**Published:** 2024-04-20

**Authors:** Lingzhi Tang, Jinmei Xia, Zhongwei Chen, Fengjiao Lin, Zongze Shao, Weiyi Wang, Xuan Hong

**Affiliations:** 1 Xiamen Key Laboratory of Marine Medicinal Natural Products Resources, Xiamen Medical College, Xiamen 361023, China; tlz@xmmc.edu.cn (L.T.); czw@xmmc.edu.cn (Z.C.); lfj03262023@163.com (F.L.); 2 Key Laboratory of Marine Biogenetic Resources, Third Institute of Oceanography, Ministry of Natural Resources, Xiamen 361005, China; xiajinmei@tio.org.cn (J.X.); shaozongze@tio.org.cn (Z.S.)

**Keywords:** *Talaromyces*, meroterpenoid, cytotoxic, antibacterial

## Abstract

Three novel meroterpenoids, taladrimanins B–D (**1**–**3**), were isolated from the marine-derived fungus *Talaromyces* sp. M27416, alongside three biogenetically related compounds (**4**–**6**). We delineated taladrimanin B’s (**1**) structure using HRESIMS and NMR, confirmed its configuration via quantum chemical NMR analysis and DP4+ methodology, and verified it through X-ray crystallography. ECD calculations determined the absolute configuration of compound **1**, while comparative NMR and ECD analyses elucidated the absolute configurations of **2** and **3**. These compounds are drimane-type meroterpenoids with a C10 polyketide unit (8*R*-configuration). We proposed a biosynthetic pathway and noted that compound **1** showed cytotoxic activity against MKN-45 and 5637 cell lines and selective antibacterial effects against *Staphylococcus aureus* CICC 10384.

## 1. Introduction

Natural products have long been recognized as a pivotal source of therapeutic agents, with a significant proportion of pharmaceuticals being derived directly or indirectly from bioactive compounds found in nature [1,2]. Among these, fungal metabolites stand out for their structural diversity and potent biological activities, which have led to the discovery of numerous drugs and drug candidates. The genus *Talaromyces* is a rich source of meroterpenoids, a class of natural products characterized by their hybrid biosynthetic origin from terpenoid and polyketide pathways [3]. These compounds exhibit a remarkable array of complex molecular architectures and a wide spectrum of biological activities, making them a focal point in the search for new pharmaceutical agents [3].

Significant meroterpenoids isolated from *Talaromyces* species include compounds such as dinapinone AB2, a compound known for its inhibitory effect on triacylglycerol synthesis in intact mammalian cells [4]. Talarodilactones A and B exhibit cytotoxic activities to the L5178Y mouse lymphoma cell line, with IC_50_ values of 3.9 and 1.3 µM, respectively [5]. These molecules are distinguished by their unique structural features, such as fused ring systems, stereochemical complexity, and functional group diversity, which underpin their biological activities [6,7]. The pharmacological potential of *Talaromyces*-derived meroterpenoids is vast, encompassing activities such as antimicrobial, anticancer, anti-inflammatory, and neuroprotective effects. The structural diversity of these compounds often correlates with their mode of action, offering insights into the relationship between structure and biological activity. This has implications not only for the development of novel therapeutics, but also for understanding the underlying principles of bioactive natural products [3].

**Figure 1:**
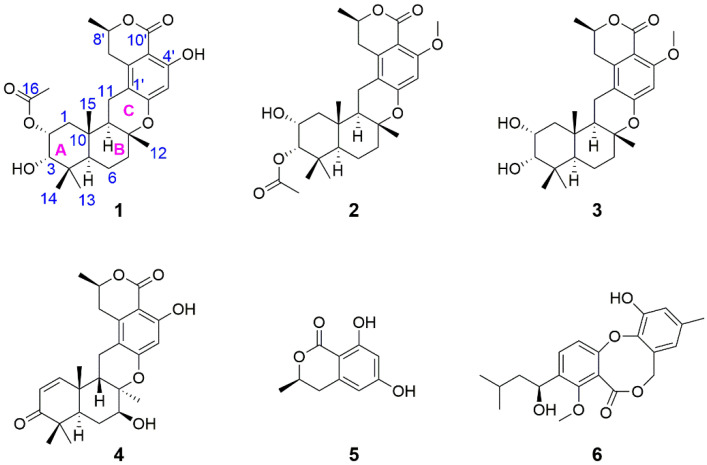
Structures of **1**–**6**.

In the course of exploring marine-derived microbial entities for bioactive substances, an intriguing finding was made concerning a *Talaromyces* sp. strain, initially designated as HM6-1-1 [8] and subsequently deposited at the Marine Culture Collection of China (MCCC) under accession number M27416. This strain was isolated from seawater samples gathered around Fujian Province’s Dongshan Island. In previous studies, the isolation of the first 8*R*-configured meroterpenoid, named taladrimanin A, was achieved [8]. Through further systematic isolation efforts, three novel meroterpenoids were obtained, named taladrimanins B–D (**1**–**3**). Accompanying these were three biogenetically linked compounds (**4**–**6**, Figure 1). Advanced spectroscopic techniques, coupled with X-ray and DFT analyses, facilitated the structural elucidation of their absolute configurations. Furthermore, biological assays were conducted to ascertain these newly identified compounds’ cytotoxic and antibacterial properties.

## 2. Results and Discussion

**Figure 2:**
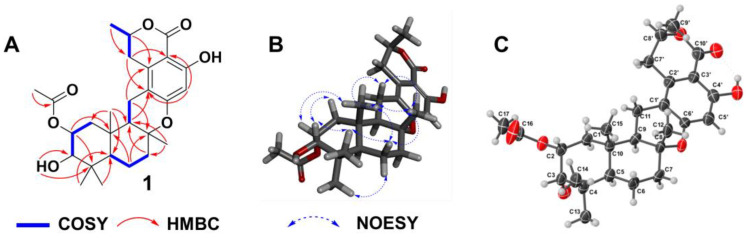
(**A**,**B**) Key HMBC, COSY, and NOESY correlations of **1**; (**C**) X-ray structure of **1**.

Taladrimanin B (**1**), obtained as an amorphous white solid, was characterized by a sodium adduct ion, *m/z* 495.2370, from which the molecular formula C_27_H_36_O_7_ was inferred, indicating a total of ten degrees of unsaturation. Spectroscopic methods including ^13^C NMR, HSQC, and ^13^C/DEPT helped in identifying various structural features (Table 1): ten quaternary carbons (at C-1′ to 4′, 6′, 10′, 4, 8, 10, 16), six methines (at C-5′, 8′, 2 to 3, 5, 9), five methylenes (at C-7′, 1, 6 to 7, 11), and six methyls (at C-9′, 12 to 15, 17). COSY correlation analysis disclosed four spin systems, C-1 to C-3, C-5 to C-7, C-9/C-11, and C-7′ to C-9′, as detailed in Figure 2. The establishment of the drimane-type sesquiterpene core was supported by various HMBCs (Figure 2), such as H-2 to C-4/C-10, H_3_-13(14) to C-5, H_2_-6 to C-8/C-10, H_2_-7 to C-9, H_3_-12 to C-7/C-9, and H_3_-15 to C-1/C-5/C-9. Other significant HMBCs (H_3_-9′ to C-7′, H-8′ to C-2′, H_2_-7′ to C-1′/C-3′, and H-5′ to C-1′/C-3′) contributed to delineating the isochroman-1-one portion with a tetra-substitution pattern (Figure 2). This moiety includes an ester group at C-10′ and two oxygenated sp^2^-hybridized quaternary carbons (C-4′ and C-6′). Linkages between sesquiterpene and isochromanone fragments, specifically through C-8-O-C-6′ and C-11-C-1′ bonds, were deduced from specific HMBC interactions (H_2_-11 to C-2′) and downfield chemical shifts of C-8 and C-6′ (Table 1; Figure 2). The positioning of the hydroxyl group at C-4′ was verified through the HMBC of OH to C-4′, as illustrated in Figure 2.

**Figure 3:**
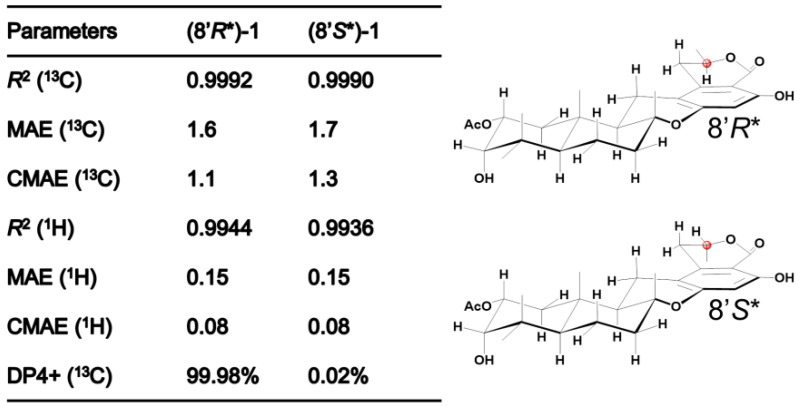
Comparison of NMR calculations of (8′R*)-**1** and (8′S*)-**1** coupled with DP4+ analysis.

**Figure 4:**
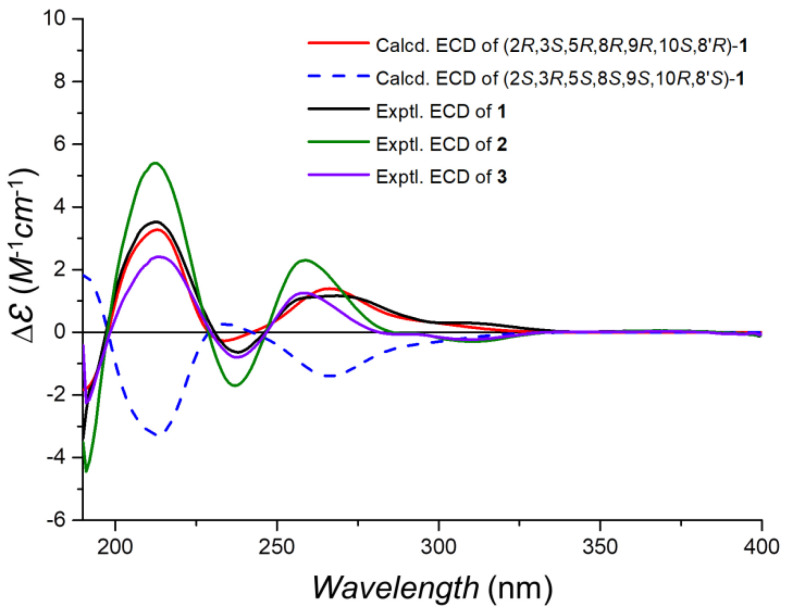
Experimental ECD of **1**–**3** and calculated ECD spectra of **1**.

The observed ^3^*J*_HH_ values, 13.1 Hz between H-9 and H-11*β* and 12.1 Hz between H-1*α* and H-2, respectively, identified that H-2, H-11*β*, and H-9 had the axial orientations. The NOESY spectrum’s cross-peaks involving H-2 with H-3 and H_3_-15, alongside H-11*β*’s interaction with H_3_-12 and the correlation between H-9 and H-11*α*, verified the *β*-orientations of H-2, H-3, and the methyl constituents at C-15 and C-12, while also confirmed H-9’s *α*-orientation. This information corroborates the configuration of rings A and B in a *trans*-decalin structure and ring C’s twist-boat conformation, as depicted in Figure 2. However, the relative configuration at C-8′ remained indeterminate, based on the NOESY and *J* coupling data, necessitating further exploration into isomers (8′R*)-1 and (8′S*)-1 through ^13^C NMR spectroscopy at the mPW1PW91/6-31+G(d,p) level. Among these, isomer (8′R*)-1 exhibited better alignment with the observed MAE and CMAE (^13^C) data, a finding supported by the DP4+ probability analysis illustrated in Figure 3. X-ray diffraction analysis on single crystals of **1** with Cu Kα radiation (Flack parameter = −0.2(3), CCDC number: 2340419) supported these spectroscopic conclusions (Figure 2). The single-crystal analysis, limited by the less-than-ideal Flack parameter, only defined the relative stereochemistry as 2R*, 3S*, 5R*, 8R*, 9R*, 10S*, and 8′R*. Following this, ECD calculations at CAM-B3LYP/6-311G(d) (Figure 4) established the absolute stereochemistry of compound **1** as 2*R*, 3*S*, 5*R*, 8*R*, 9*R*, 10*S*, and 8′*R*.

**Figure 5:**
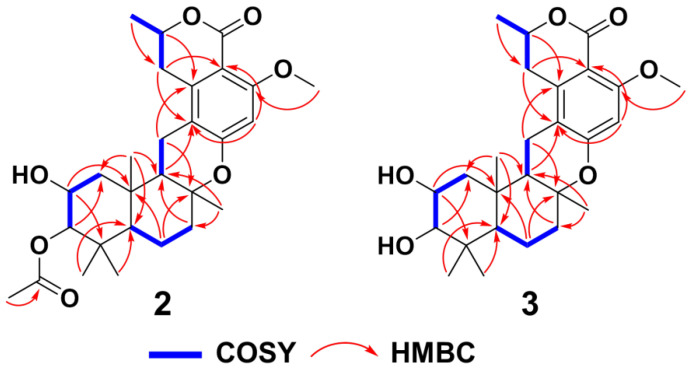
Key COSY and HMBCs of **2** and **3**.

Taladrimanin C (**2**), obtained as a white amorphous solid and exhibited a sodium adduct ion of *m/z* 509.2521, provided a molecular formula of C_28_H_38_O_7_, indicating ten degrees of unsaturation. The molecular weight of **2** was 14 units greater than that of **1**, suggesting the addition of a methyl group in its structure. By comparing the NMR data of **2** and **1**, it was determined that their planar structures were very similar, with the primary difference located at C-1, C-2, C-3, and C-10′, with **2** additionally featuring a methoxy signal at C-11′ (Table 1). The COSY correlations of C1 to C3 (Figure 5) and the downfield chemical shifts of C-2 and 3 indicated that C-2 and C-3 were O-bearing, and an acetyl group was attached via an oxygen atom at C-3. Additionally, HMBCs from H_3_-11′ to C-4′ confirmed the position of the methoxy group that **2** had more than **1** (Figure 5). The HMBCs closely matched those of **1**, enabling the clear identification of the sesquiterpene and isochromanone units. These units were fused via C-8-O-C-6′ and C-11-C-1′ linkages (Figure 5). A careful comparison of the NOESY correlation signals of **2** and **1** established that they shared the same relative configuration. The similarity of their ECD curves suggested they had identical absolute configurations (Figure 4). Hence, the absolute configuration of **2** was 2*R*, 3*S*, 5*R*, 8*R*, 9*R*, 10*S*, and 8′*R*.

Taladrimanin D (**3**), a white amorphous solid with a sodium adduct ion of *m/z* 467.2411, has a molecular formula of C_26_H_36_O_6_, requiring nine degrees of unsaturation. The molecular weight is 42 Da lower than that of **2**, suggesting it likely lacks an acetyl group. NMR analysis showed that **3** and **2** have similar planar structures (Table 1), with key differences in C-2 and C-5. COSY correlations from C1 to C3 (Figure 5) and downfield chemical shifts at C-2 and C-3 suggest that hydroxyl groups are attached to both C-2 and C-3. Similarly, the HMBC signals for **3** closely resembled those of compounds **1** and **2**, reinforcing the similarity in their planar structures. NOESY signal comparison across **1**, **2**, and **3** confirms their identical relative configurations. ECD curve similarities for **1**, **2**, and **3** (Figure 4) indicate a shared absolute configuration, confirming compound **3**’s configuration as 2*R*, 3*S*, 5*R*, 8*R*, 9*R*, 10*S*, and 8′*R*.

**Figure 6:**
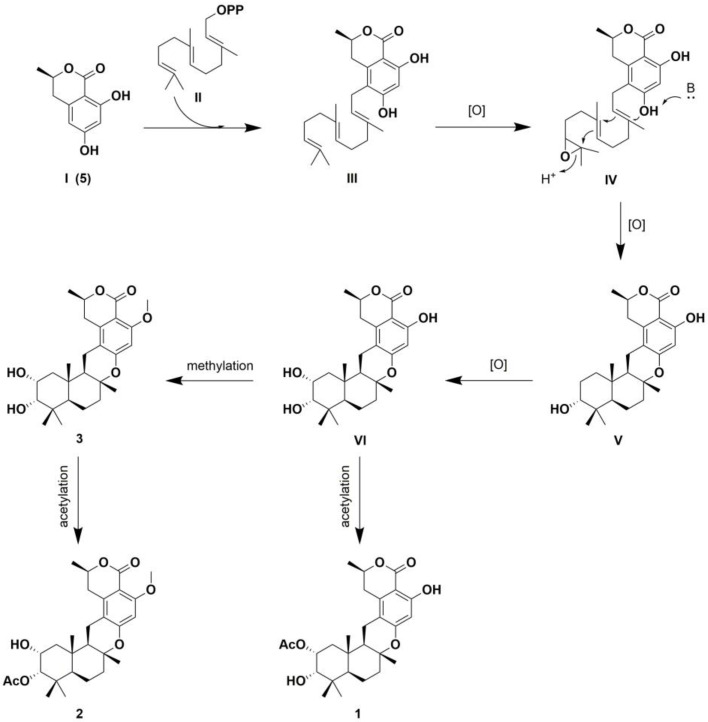
Postulated biogenetic pathway of **1**–**3**.

Compounds **1**–**3** were supposed to be derived from C10 polyketone 6-hydroxymellein (**5**), undergoing prenylation, cyclization, oxidation, acetylation, or methylation (Figure 6). Unlike chrodrimanins, pentacecilides, and verruculides, compounds **1**–**3** uniquely feature an *R* configuration at C-8. NMR data comparison with the literature identified compounds **4**–**6** as chrodrimanin E (**4**) [9], (*R*)-6-hydroxymellein (**5**) [10], and penicillide (**6**) [11], respectively.

**Figure 7:**
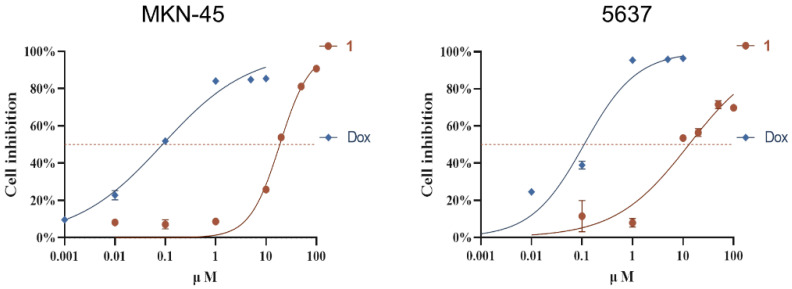
Compound **1** inhibited the proliferation of MKN-45 and 5637 cells in the CCK8 assay.

Cytotoxicity tests of compounds **1**–**4** on twenty cancer cell lines showed that compound **1** inhibited MKN-45 and 5637 cells by 52.26% and 53.41% at 20 µΜ, respectively. Further analysis showed IC_50_ values of 18.8 µΜ for MKN-45 and 13.0 µΜ for 5637 cells, with doxorubicin (Dox) as a positive control (IC_50_: 0.09 µΜ and 0.11 µΜ, respectively) (Figure 7). Antibacterial testing of compound **1** against various strains revealed selective activity against *Staphylococcus aureus* CICC 10384, with an MIC of 12.5 µg/mL (chloramphenicol as a positive control with an MIC of 5.0 µg/mL).

## 3. Materials and Methods

### 3.1. General Experimental Procedures

The methodology utilized in this investigation has been outlined in prior studies [12]. Data acquisition via X-ray was executed using a Bruker APEX-II CCD diffractometer. The fungal species *Talaromyces* sp. M27416 underwent characterization through ITS sequence analysis, with the sequence being archived in GenBank under the accession number OL744613.

### 3.2. Fermentation, Extraction, and Isolation

The methodology for the fermentation and subsequent extraction process has been detailed in the extant literature [12]. Utilizing silica gel (200–300 mesh) for chromatography, the dry extract weighing 80 g underwent elution with a gradient of PE/EtOAc (from 100:0 to 0:100), followed by an EtOAc/MeOH gradient (also from 100:0 to 0:100). This process resulted in the collection of ten fractions (Fraction 1 to Fraction 10). Fraction 5 was further processed using Sephadex LH-20 chromatography (in MeOH), leading to the isolation of four subfractions (Fraction 5-1 to Fraction 5-4). The purification of Fraction 5-4 via ODS chromatography resulted in the isolation of **5** (4.8 mg). Similarly, Fraction 6 was divided into four subfractions (Fraction 6-1 to Fraction 6-4) through Sephadex LH-20 chromatography (in MeOH). Subsequent purification of Fraction 6-4 through ODS chromatography yielded **6** (4.3 mg), **4** (3.1 mg), and **1** (3.9 mg). Finally, Fraction 10 was separated into two subfractions (Fraction 10-1 and Fraction 10-2) using Sephadex LH-20 (in MeOH), with Fraction 10-1 undergoing ODS purification to obtain **2** (4.1 mg) and **3** (1.6 mg). ODS chromatography was conducted under the following conditions: a gradient elution starting from a 20% acetonitrile–water solution to 80% acetonitrile–water solution over 20 min; followed by a transition from 80% acetonitrile–water solution to 100% acetonitrile within 5 min; and finally, elution with 100% acetonitrile for 15 min.

Taladrimanin B (**1**): white amorphous solid; ${\left[\alpha \right]}_{D}^{25.0}24.0\left(c\;0.1,MeOH\right);$ UV λ_max_ (methanol) nm (log *ε*): 220 (3.93), 275 (3.72), 310 (3.28); NMR data are shown in Table 1; HR-ESI-MS: *m*/*z* 495.2370 [M + Na]^+^ (Calcd. for 495.2359, C_27_H_36_NaO_7_, ∆ + 2.2 ppm).

Taladrimanin C (**2**): white amorphous solid; ${\left[\alpha \right]}_{D}^{25.0}21.9\left(c\;0.1,MeOH\right);$ UV λ_max_ (methanol) nm (log *ε*): 216 (4.06), 270 (3.71), 310 (3.46); NMR data are shown in Table 1; HR-ESI-MS: *m*/*z* 509.2521 [M + Na]^+^ (Calcd. for 509.2515, C_28_H_38_NaO_7_, ∆ + 1.2 ppm).

Taladrimanin D (**3**): white amorphous solid; ${\left[\alpha \right]}_{D}^{25.0}7.2\left(c\;0.1,MeOH\right);$ UV λ_max_ (methanol) nm (log *ε*): 216 (3.91), 270 (3.55), 310 (3.31); NMR data are shown in Table 1; HR-ESI-MS: *m*/*z* 467.2411 [M + Na]^+^ (Calcd. for 467.2410, C_26_H_36_NaO_6_, ∆ + 0.2 ppm).

X-ray crystallography for Taladrimanin B (**1**): C_27_H_36_O_7_ (*M* = 472.56 g/mol): monoclinic, space group C2 (no. 5), *a* = 40.456(3) Å, *b* = 6.4092(4) Å, *c* = 19.6227(15) Å, *β* = 108.559(6), *V* = 4823.4(6) Å^3^, *Z* = 8, *T* = 100 K, µ(CuKα) = 0.759 mm^−1^, *Dcalc* = 1.301 g/cm^3^, 22,909 reflections measured (4.608° ≤ 2Θ ≤ 149.456°), 9110 unique (*R*_int_ = 0.1348, R_sigma_ = 0.1298), which were used in all calculations. The final *R*_1_ was 0.0701 (I > 2σ(I)) and *wR*_2_ was 0.1842 (all data).

### 3.3. NMR Calculation

For the conformational analysis, Crest software (V 2.11) was employed [13]. Density functional theory (DFT) calculations were conducted utilizing the Gaussian 16 software [14]. Comprehensive details on the computational approach and the DP4+ analysis procedure are available in the Supplementary Materials [12,15]. Time-dependent density functional theory (TDDFT) ECD calculations were computed at CAM-B3LYP/6-311G(d) level of theory in MeOH with the IEFPCM solvent model. For each conformer, 36 excited states were calculated [16]. The calculated ECD curves were generated using Multiwfn 3.6 software [17].

### 3.4. Cytotoxic and Antibacterial Assay

The cancer cell lines A549, MKN-45, HCT 116, HeLa, K-562, 786-O, TE-1, 5637, GBC-SD, L-02, MCF7, HepG2, SF126, DU145, CAL-62, PATU8988T, HOS, A-375, A-673, and 293T were obtained from Wuhan Pricella Biotechnology Co., Ltd. (Wuhan, China). These cell lines were cultured according to previously established protocols [18]. The CCK8 assays were used to determine the impact of compounds on the viability of cancer cells, adhering to previously described methods [18]. Moreover, the protocol from earlier studies [19,20] was replicated to conduct the antibacterial assays using *Staphylococcus aureus* (CICC 10384), *Escherichia coli* (CICC 10302), *Vibrio parahaemolyticus* (vp-HL) [21], *Vibrio parahaemolyticus* (ATCC 17802), *Vibrio vulnificus* (MCCC 1A08743), and *Salmonella enteritidis* (CICC 21482) as tested strains.

## 4. Conclusions

In this study, we obtained three meroterpenoids (compounds **1**–**3**) and three biogenetically related compounds (**5**–**6**) and elucidated their structures. We specifically examined compound **1** using NMR computation and DP4+ analysis to determine the absolute configuration at the chiral center C-8′. X-ray crystallography confirmed the chirality, validating the effectiveness of DP4+ in the structural elucidation of natural products. Our findings reveal that meroterpenoids **1**–**3** belong to the drimane-type and feature a C10 polyketide backbone with an 8*R* configuration, setting them apart from similar polyketide-terpenoids such as chrodrimanins, pentacecilides, and verruculides. Significantly, compound **1** demonstrated a capacity to reduce cell viability in human cancer cell lines MKN-45 and 5637, and exhibited a selective antibacterial effect against *S. aureus*. These results highlight the potential of structural modifications in fungal meroterpenoids for developing lead compounds and suggest new directions for future research.

## Figures and Tables

**Table 1:** ^1^H NMR data (850 MHz) and ^13^C NMR data (214 MHz) for compounds **1**–**3** (chloroform-*d*).

	1		2		3	
Position	*δ*_C_, Type	*δ*_H_, Mult. (*J* in Hz)	*δ*_C_, Type	*δ*_H_, Mult. (*J* in Hz)	*δ*_C_, Type	*δ*_H_, Mult. (*J* in Hz)
1-*β*	36.9, CH_2_	1.80 (dd, 11.7, 4.5 Hz, 1H)	41.0, CH_2_	1.88 (dd, 12.1, 4.4 Hz, 1H)	40.8, CH_2_	1.82 (dd, 11.9, 4.6 Hz, 1H)
1-*α*	36.9, CH_2_	1.58 (overlapped ^a^, 1H)	41.0, CH_2_	1.39 (t, 12.1 Hz, 1H)	40.8, CH_2_	1.42 (overlapped ^a^, 1H)
2-*β*	70.9, CH	5.25 (ddd, 12.1, 4.4, 2.5 Hz, 1H)	65.7, CH	4.23 (ddd, 12.0, 4.4, 2.9 Hz, 1H)	66.3, CH	4.08 (dt, 11.9, 4.1 Hz, 1H)
3-*β*	76.5, CH	3.53 (brs, 1H)	80.3, CH	4.93 (d, 2.8 Hz, 1H)	78.7, CH	3.48 (d, 2.9 Hz, 1H)
4	38.5, C	-	38.1, C	-	37.9, C	-
5	47.9, CH	1.64 (d, 12.6, 1H)	49.5, CH	1.47 (overlapped ^a^, 1H)	47.9, CH	1.53 (overlapped ^a^, 1H)
6-*α*	18.9, CH_2_	1.72 (overlapped ^a^, 1H)	18.9, CH_2_	1.72 (overlapped ^a^, 1H)	19.0, CH_2_	1.72 (overlapped ^a^, 1H)
6-β	18.9, CH_2_	1.40 (dd, 12.8, 3.4, 1H)	18.9, CH_2_	1.42 (dd, 13.4, 3.4, 1H)	19.0, CH_2_	1.40 (overlapped ^a^, 1H)
7-*β*	40.4, CH_2_	2.09 (overlapped ^a^, 1H)	40.5, CH_2_	2.14 (dt, 13.6, 3.5 Hz, 1H)	40.6, CH_2_	2.11 (dt, 11.2, 3.0 Hz, 1H)
7-*α*	40.4, CH_2_	1.72 (overlapped ^a^, 1H)	40.5, CH_2_	1.75 (overlapped ^a^, 1H)	40.6, CH_2_	1.71 (overlapped ^a^, 1H)
8	77.9, C	-	77.7, C	-	77.8, C	-
9	51.2, CH	1.76 (dd, 13.1, 5.1 Hz, 1H)	51.6, CH	1.78 (dd, 13.1, 5.0 Hz, 1H)	51.5, CH	1.75 (m, 1H)
10	38.0, C	-	37.8, C	-	38.3, C	-
11-*α*	19.4, CH_2_	2.48 (dd, 15.9, 5.1 Hz, 1H)	19.3, CH_2_	2.60 (m, 1H)	19.4, CH_2_	2.59 (m, 1H)
11-*β*	19.4, CH_2_	2.25 (dd, 15.8, 13.1 Hz, 1H)	19.3, CH_2_	2.29 (dd, 15.8, 13.2 Hz, 1H)	19.4, CH_2_	2.28 (dd, 15.8, 13.1 Hz, 1H)
12	21.0, CH_3_	1.17 (s, 3H)	21.0, CH_3_	1.20 (s, 3H)	20.9, CH_3_	1.18 (s, 3H)
13	28.4, CH_3_	1.07 (s, 3H)	28.1, CH_3_	0.95 (s, 3H)	28.5, CH_3_	1.07 (s, 3H)
14	21.7, CH_3_	0.95 (s, 3H)	21.7, CH_3_	1.00 (s, 3H)	21.6, CH_3_	0.91 (s, 3H)
15	15.8, CH_3_	1.01 (s, 3H)	16.0, CH_3_	0.99 (s, 3H)	15.9, CH_3_	0.96 (s, 3H)
16	170.4, C	-	172.1, C	-		
17	21.4, CH_3_	2.11 (s, 3H)	21.0, CH_3_	2.11 (s, 3H)		
1′	110.7, C	-	110.2, C	-	110.3, C	-
2′	139.1, C	-	141.8, C	-	141.8, C	-
3′	101.7, C	-	106.6, C	-	106.5, C	-
4′	162.2, C	-	161.5, C	-	161.5, C	-
5′	103.4, CH	6.28 (s, 1H)	99.7, CH	6.33 (s, 1H)	99.8, CH	6.33 (s, 1H)
6′	160.4, C	-	158.5, C	-	158.6, C	-
7′a	31.4, CH_2_	2.95 (dd, 16.7, 3.4 Hz, 1H)	32.3, CH_2_	2.87 (dd, 16.4, 2.8 Hz, 1H)	32.4, CH_2_	2.88 (dd, 16.4, 2.8 Hz, 1H)
7′b	31.4, CH_2_	2.61 (dd, 16.8, 11.5 Hz, 1H)	32.3, CH_2_	2.57 (dd, 14.4, 9.5 Hz, 1H)	32.4, CH_2_	2.57 (m, 1H)
8′	74.7, CH	4.63 (dqd, 12.8, 6.3, 3.0 Hz, 1H)	72.8, CH	4.45 (ddd, 9.0, 6.1, 2.7 Hz, 1H)	72.8, CH	4.48 (dtt, 12.7, 6.5, 3.2 Hz, 1H)
9′	20.9, CH_3_	1.55 (d, 6.2 Hz, 3H)	20.8, CH_3_	1.49 (d, 6.1 Hz, 3H)	20.8, CH_3_	1.49 (d, 6.3 Hz, 3H)
10′	170.1, C	-	163.1, C	-	163.2, C	-
11′			56.1, CH_3_	3.86 (s, 3H)	56.0, CH_3_	3.86 (s, 3H)
OH-4′	-	11.09 (s, 1H)				

^a^ Overlapped with the signal of other protons.

## Data Availability

The original data presented in the study are included in the article/Supplementary Materials; further inquiries can be directed to the corresponding author.

## Supplementary Materials

The following supporting information can be downloaded at: https://www.mdpi.com/article/10.3390/md22040186/s1, NMR spectra for compounds **1**–**3** as well as computational data for (8′*R**)-**1** and (8′*S**)-**1** are in Figures S1–S25 and Tables S1–S17.
