# Supplementary material for: Cytotoxic and Antibacterial Meroterpenoids Isolated from the Marine-Derived Fungus Talaromyces sp. M27416
Source: Mar Drugs. 2024 Apr 20;22(4):186. doi: 10.3390/md22040186 (PMC11051162; doi:10.3390/md22040186)
Supplement: Supplementary file 1 [file marinedrugs-22-00186-s001.zip › marinedrugs-2954260-supplementary.pdf]

Cytotoxic and Antibacterial Meroterpenoids Isolated from the Marine-Derived Fungus *Talaromyces* sp. M27416

Lingzhi Tang [1,#], Jinmei Xia [2,#] , Zhongwei Chen [1], Fengjiao Lin [1], Zongze Shao [2], Weiyi Wang [2,*], Xuan Hong [1,*]

Table of Contents

Figure S1. MS spectrum of 1 3
Figure S2. ^{1}H NMR spectrum of 1 3
Figure S3. ^{13}C NMR spectrum of 1 4
Figure S4. DEPT spectrum of 1 5
Figure S5. HSQC spectrum of 1 6
Figure S6. HMBC spectrum of 1 7
Figure S7. COSY spectrum of 1 8
Figure S8. NOESY spectrum of 1 9
Figure S9. MS spectrum of 2 9
Figure S10. ^{1}H NMR spectrum of 2 10
Figure S11. ^{13}C NMR spectrum of 2 11
Figure S12. DEPT spectrum of 2 12
Figure S13. HSQC spectrum of 2 13
Figure S14. HMBC spectrum of 2 14
Figure S15. COSY spectrum of 2 15
Figure S16. NOESY spectrum of 2 16
Figure S17. MS spectrum of 3 16
Figure S18. ^{1}H NMR spectrum of 3 17
Figure S19. ^{13}C NMR spectrum of 3 18
Figure S20. DEPT spectrum of 3 19
Figure S21. HSQC spectrum of 3 20
Figure S22. HMBC spectrum of 3 21
Figure S23. COSY spectrum of 3 22
Figure S24. NOESY spectrum of 3 23
Table S1. Experimental and calculated ^{13}C-NMR chemical shifts of 8'R-1 24
Table S2. Experimental and calculated ^{1}H-NMR chemical shifts of 8'R-1 24
Table S3. Conformational analysis of the B3LYP/6-31G(d) optimized conformers of 8'R-1 in the gas phase (T=298.15 K) 25
Table S4. Atomic coordinates (Å) of 8'R-1-1 obtained at the B3LYP/6-31G(d) level of theory in the gas phase. 25
Table S5. Atomic coordinates (Å) of 8'R-1-2 obtained at the B3LYP/6-31G(d) level of theory in the gas phase. 26
Table S6. Atomic coordinates (Å) of 8'R-1-3 obtained at the B3LYP/6-31G(d) level of theory in the gas phase. 26
Table S7. Atomic coordinates (Å) of 8'R-1-4 obtained at the B3LYP/6-31G(d) level of theory in the gas phase. 27
Table S8. Experimental and calculated ^{13}C-NMR chemical shifts of 8'S-1 28
Table S9. Experimental and calculated ^{1}H-NMR chemical shifts of 8'S-1 28
Table S10. Conformational analysis of the B3LYP/6-31G(d) optimized conformers of 8'S-1 in the gas phase (T=298.15 K) 29
Table S11. Atomic coordinates (Å) of 8'S-1-1 obtained at the B3LYP/6-31G(d) level of theory in the gas phase. 29
Table S12. Atomic coordinates (Å) of 8'S-1-2 obtained at the B3LYP/6-31G(d) level of theory in the gas phase. 30
Table S13. Atomic coordinates (Å) of 8'S-1-3 obtained at the B3LYP/6-31G(d) level of theory in the gas phase. 30

Figure S25. Result of DP4+ analysis (Isomer 1: 8’R-1; Isomer 2: 8’S-1) 31
Table S14. Key transitions, oscillator strengths, and rotatory strengths in the ECD spectrum of conformer 8’R-1-1 at the CAM-B3LYP/6-311G(d) level of theory in MeOH with IEFPCM solvent model. 31
Table S15. Key transitions, oscillator strengths, and rotatory strengths in the ECD spectrum of conformer 8’R-1-2 at the CAM-B3LYP/6-311G(d) level of theory in MeOH with IEFPCM solvent model. 33
Table S16. Key transitions, oscillator strengths, and rotatory strengths in the ECD spectrum of conformer 8’R-1-3 at the CAM-B3LYP/6-311G(d) level of theory in MeOH with IEFPCM solvent model. 35
Table S17. Key transitions, oscillator strengths, and rotatory strengths in the ECD spectrum of conformer 8’R-1-4 at the CAM-B3LYP/6-311G(d) level of theory in MeOH with IEFPCM solvent model. 37

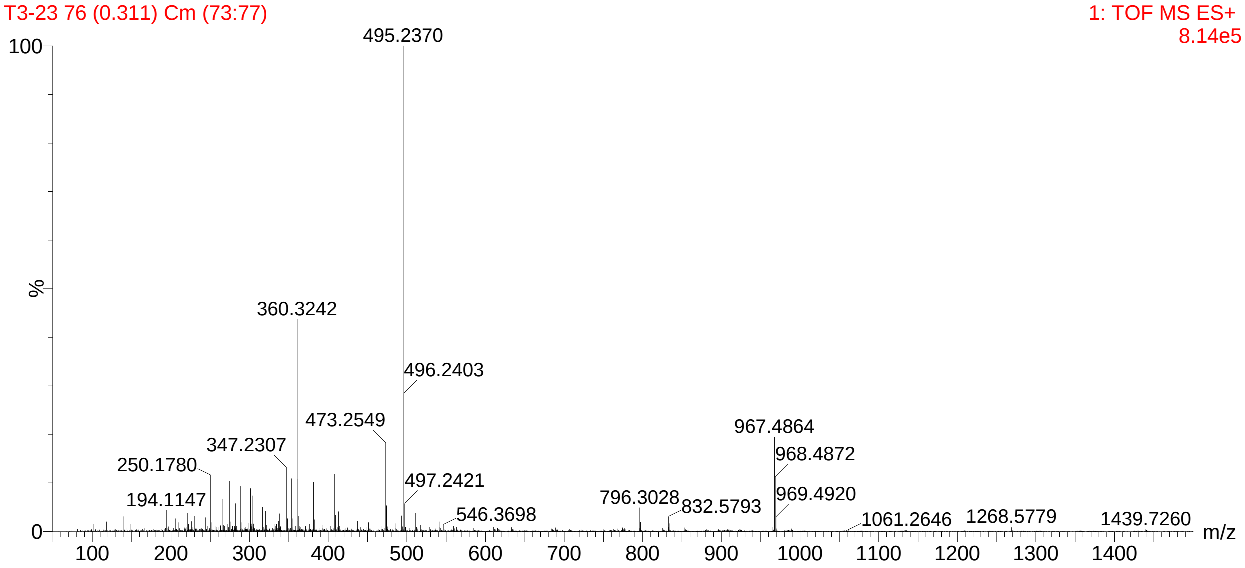

Figure S1. MS spectrum of **1**

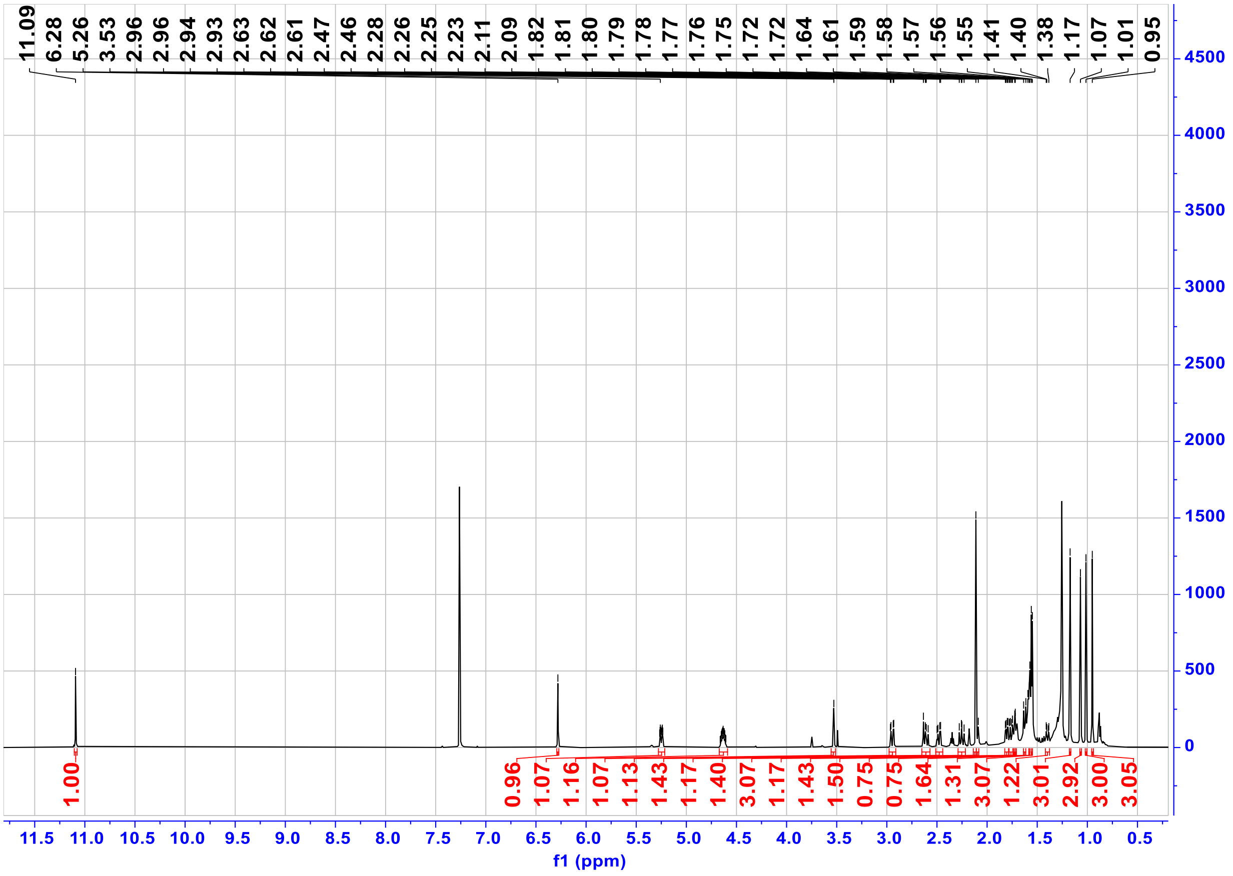

Figure S2. ^{1}H NMR spectrum of **1**

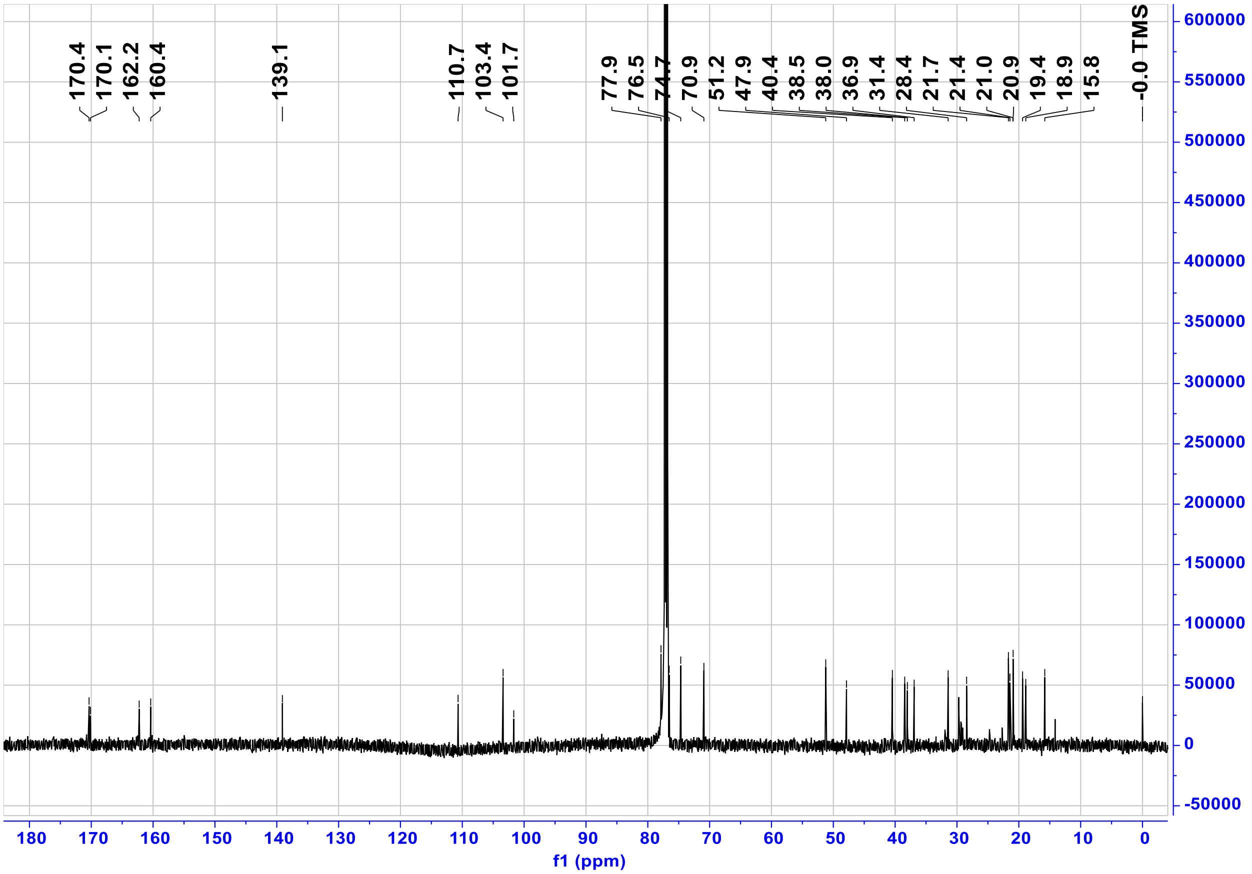

Figure S3. ^{13}C NMR spectrum of **1**

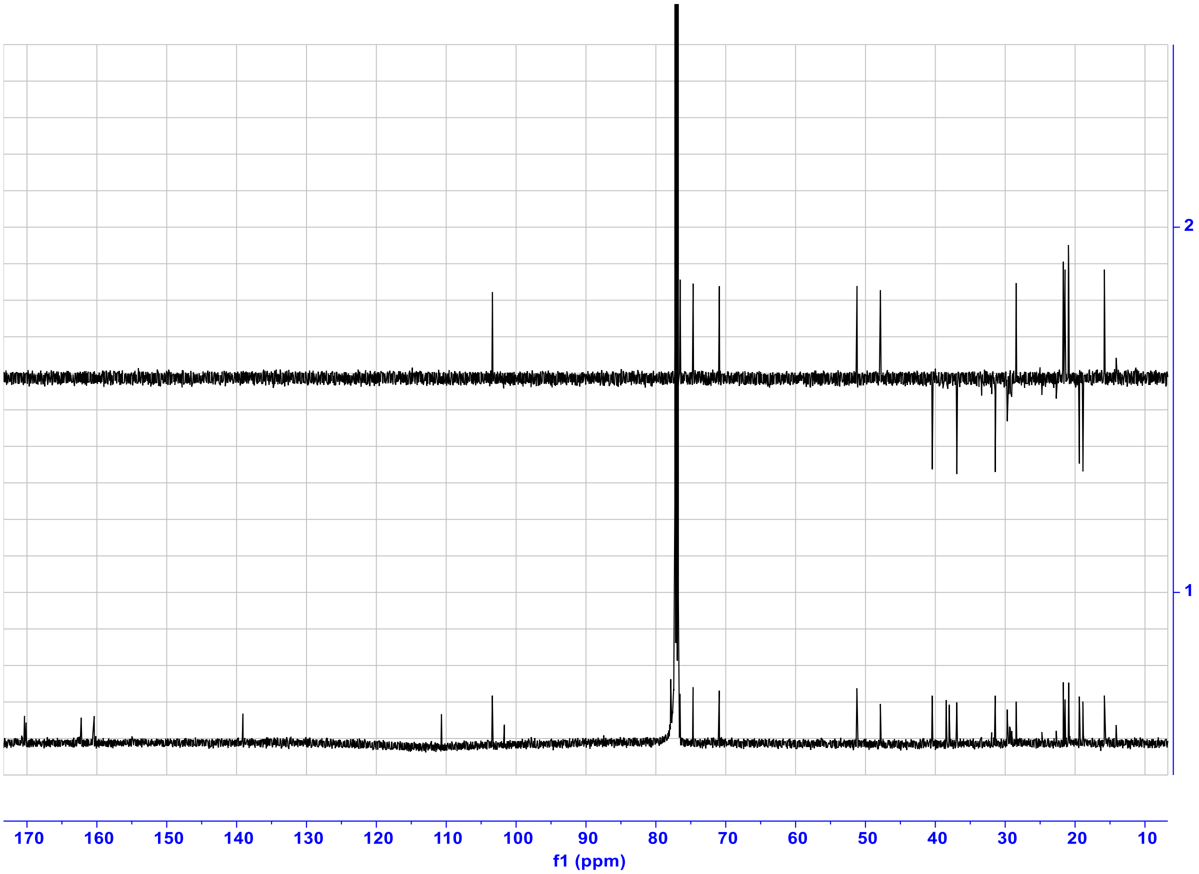

Figure S4. DEPT spectrum of **1**

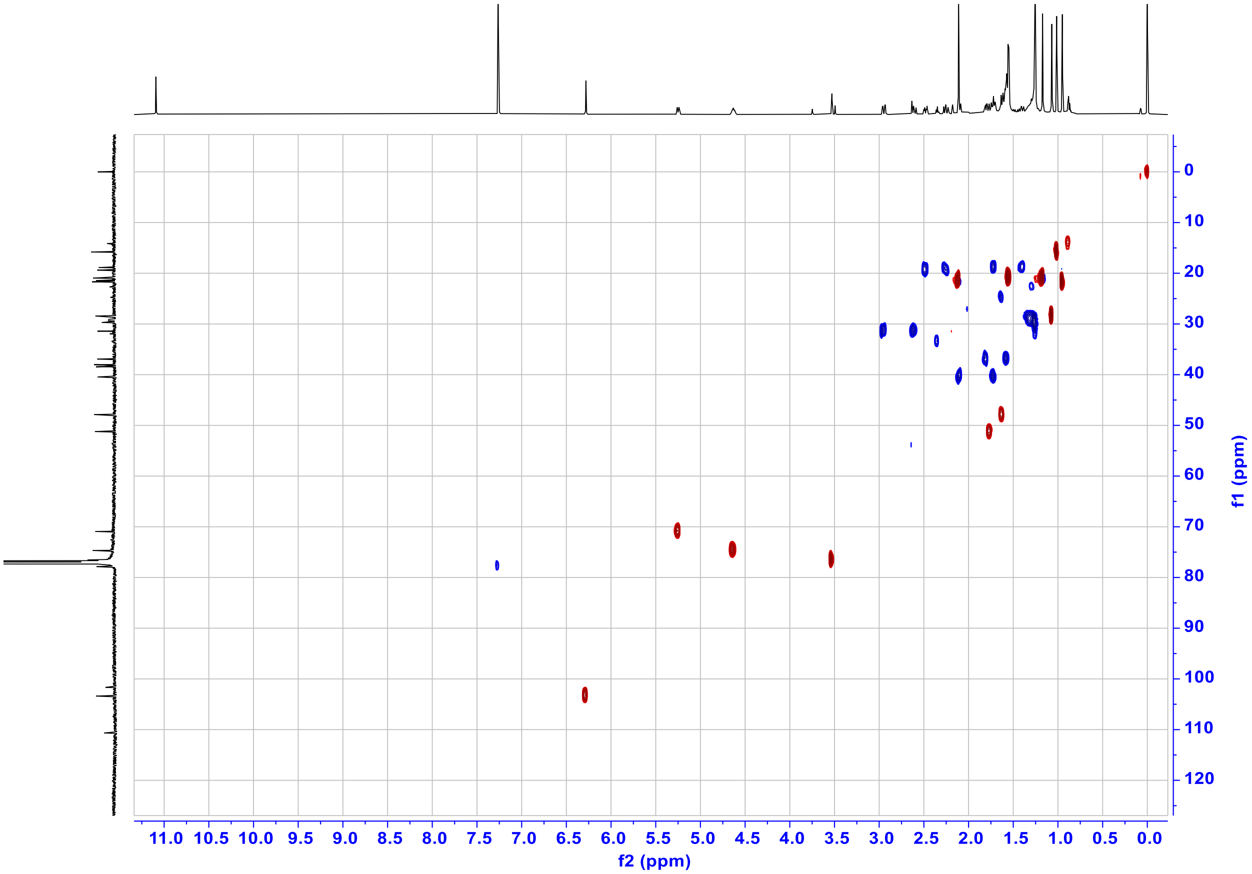

Figure S5. HSQC spectrum of **1**

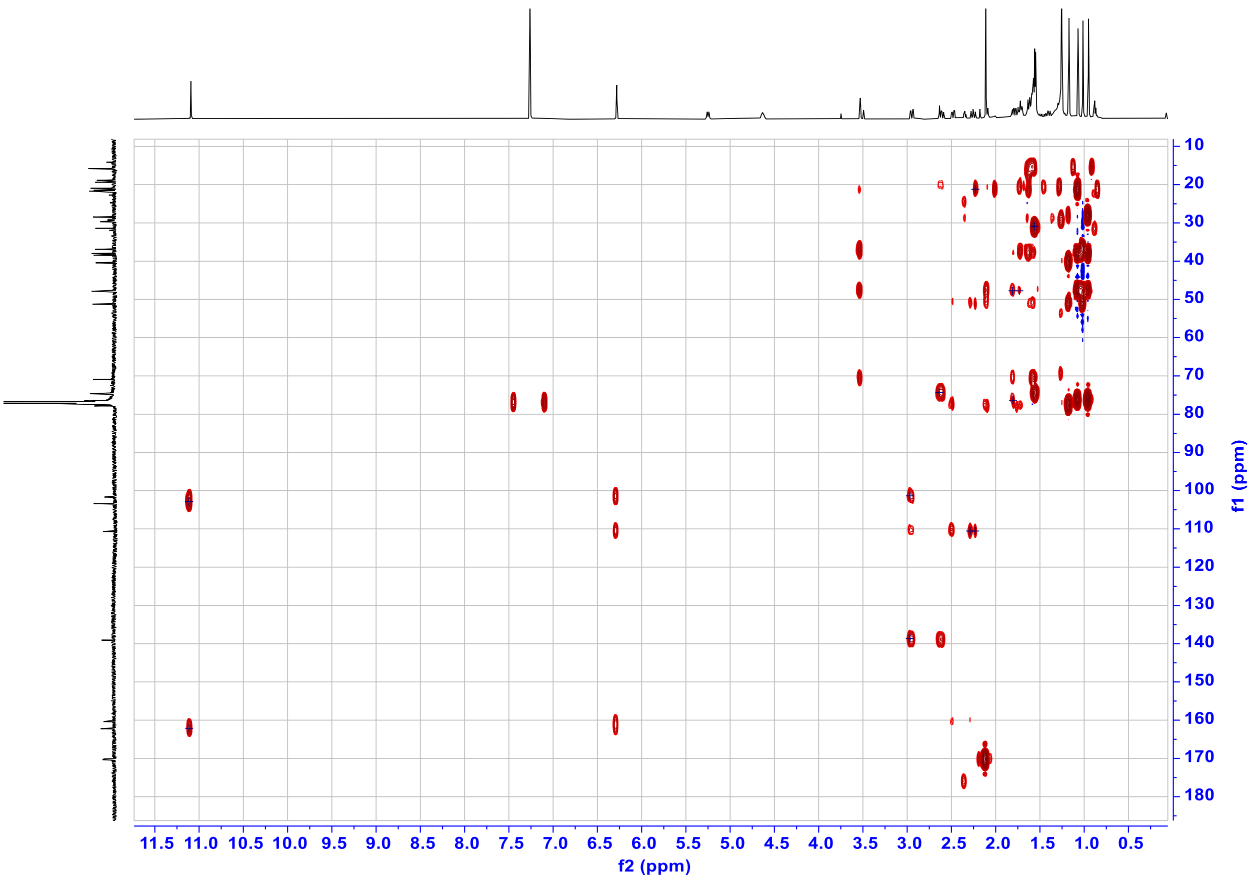

Figure S6. HMBC spectrum of **1**

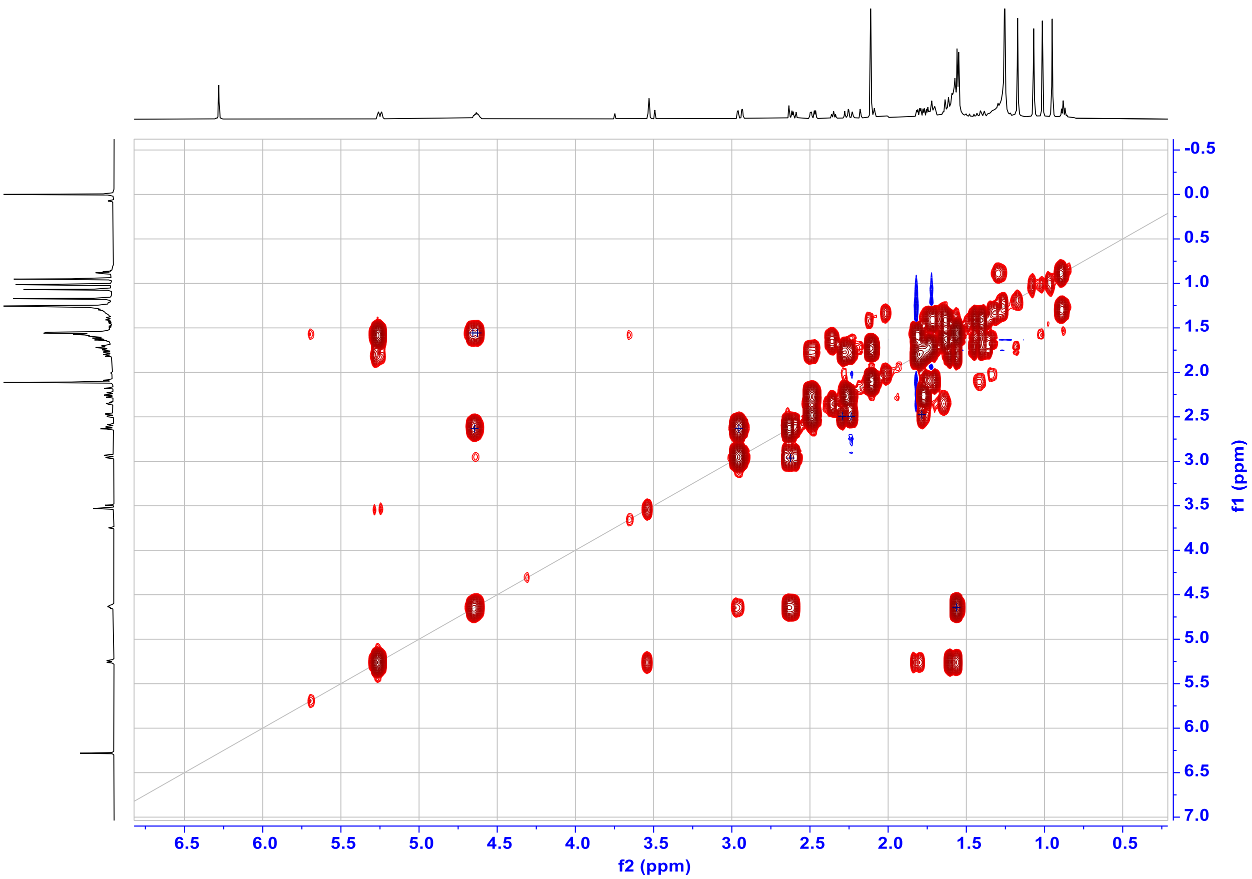

Figure S7. COSY spectrum of **1**

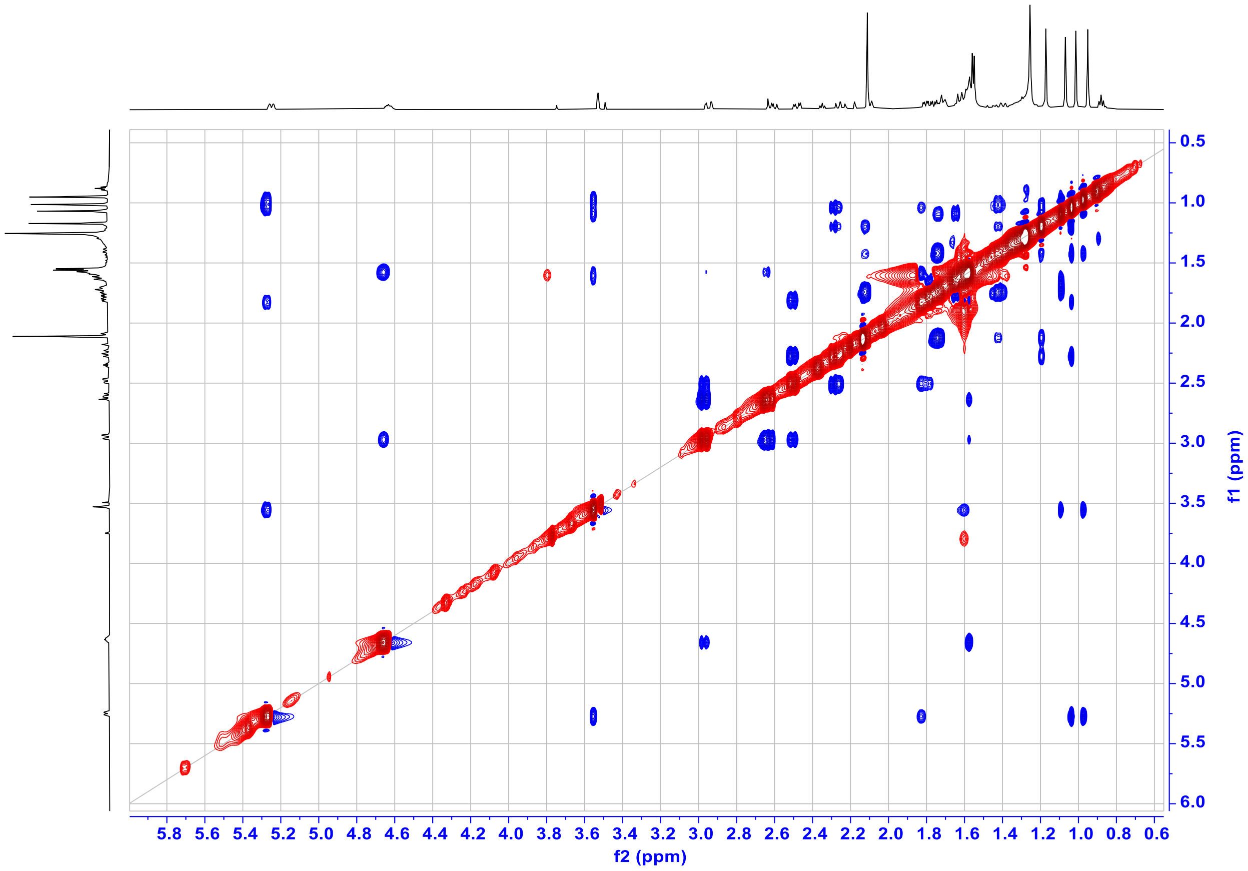

Figure S8. NOESY spectrum of **1**

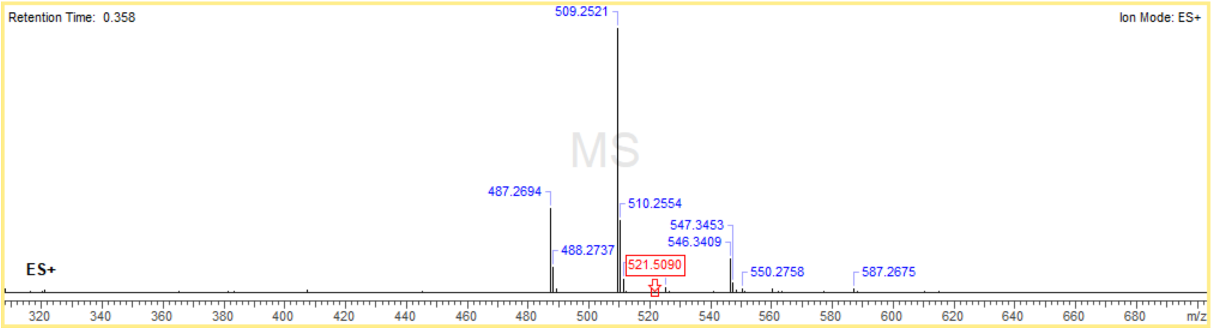

Figure S9. MS spectrum of **2**

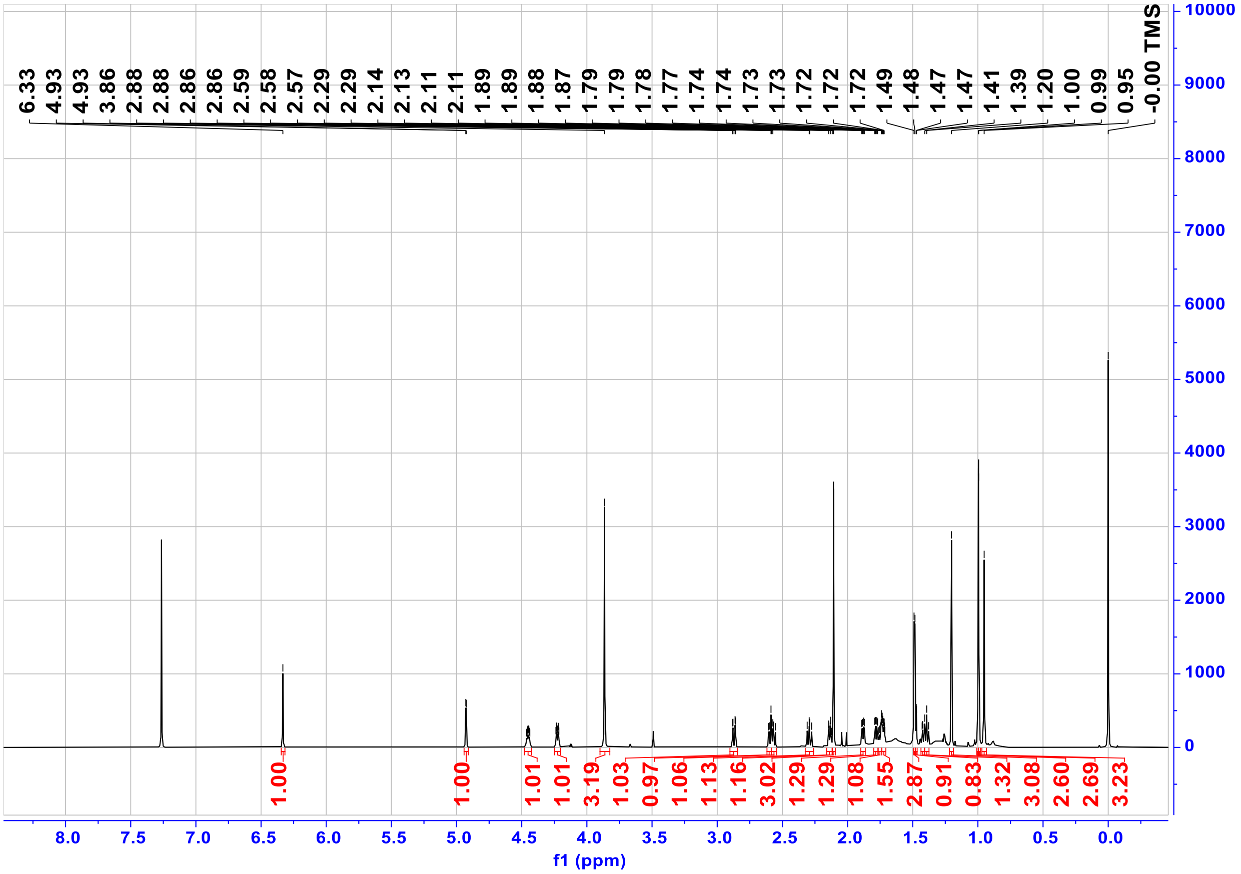

Figure S10. ^{1}H NMR spectrum of **2**

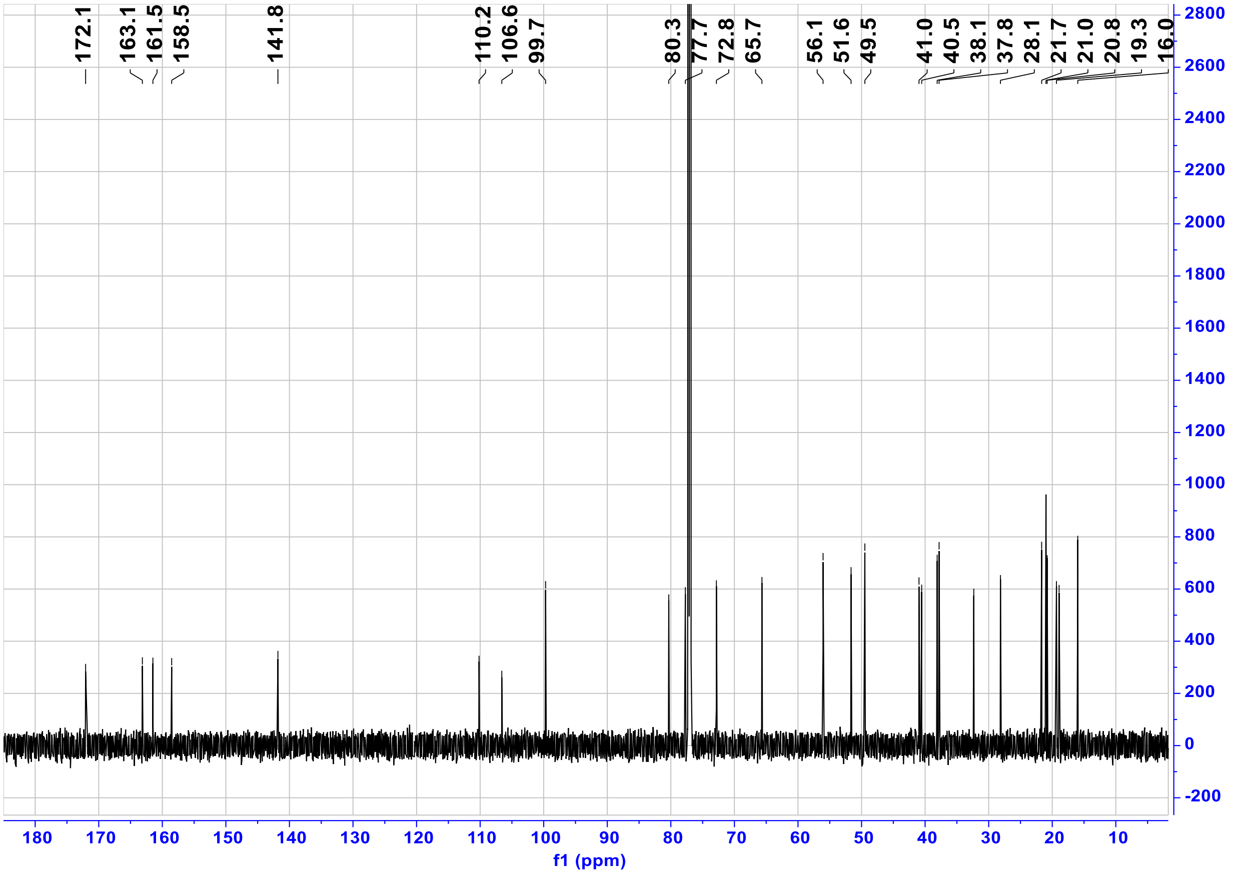

Figure S11. ^{13}C NMR spectrum of **2**

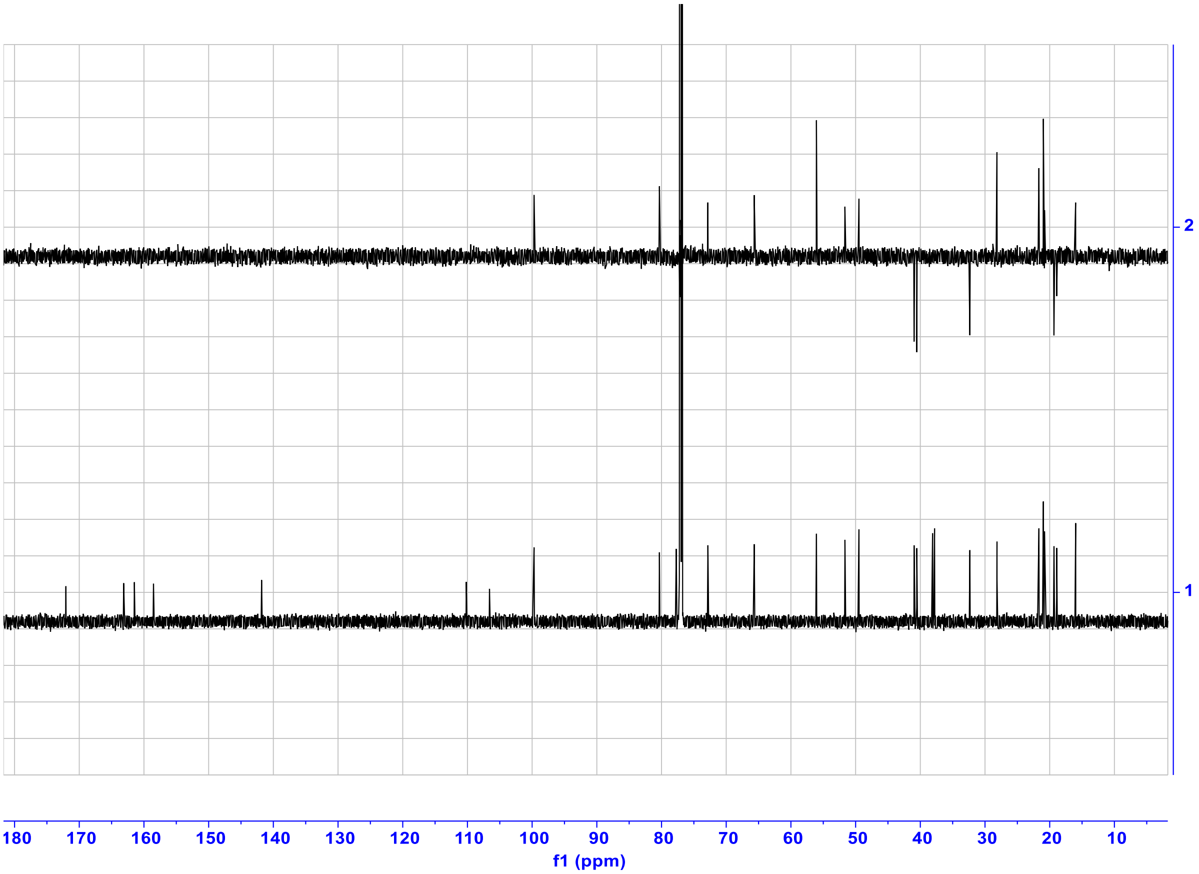

Figure S12. DEPT spectrum of **2**

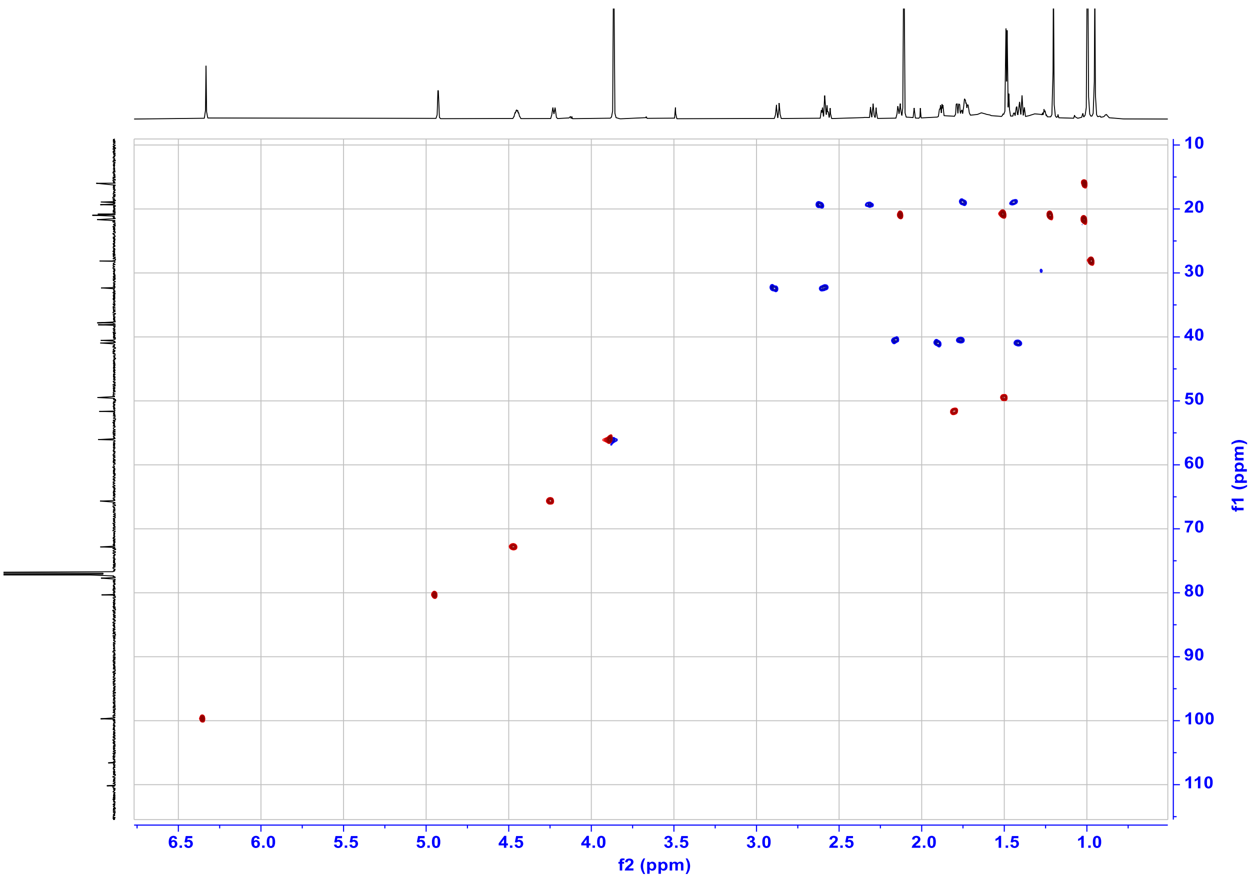

Figure S13. HSQC spectrum of **2**

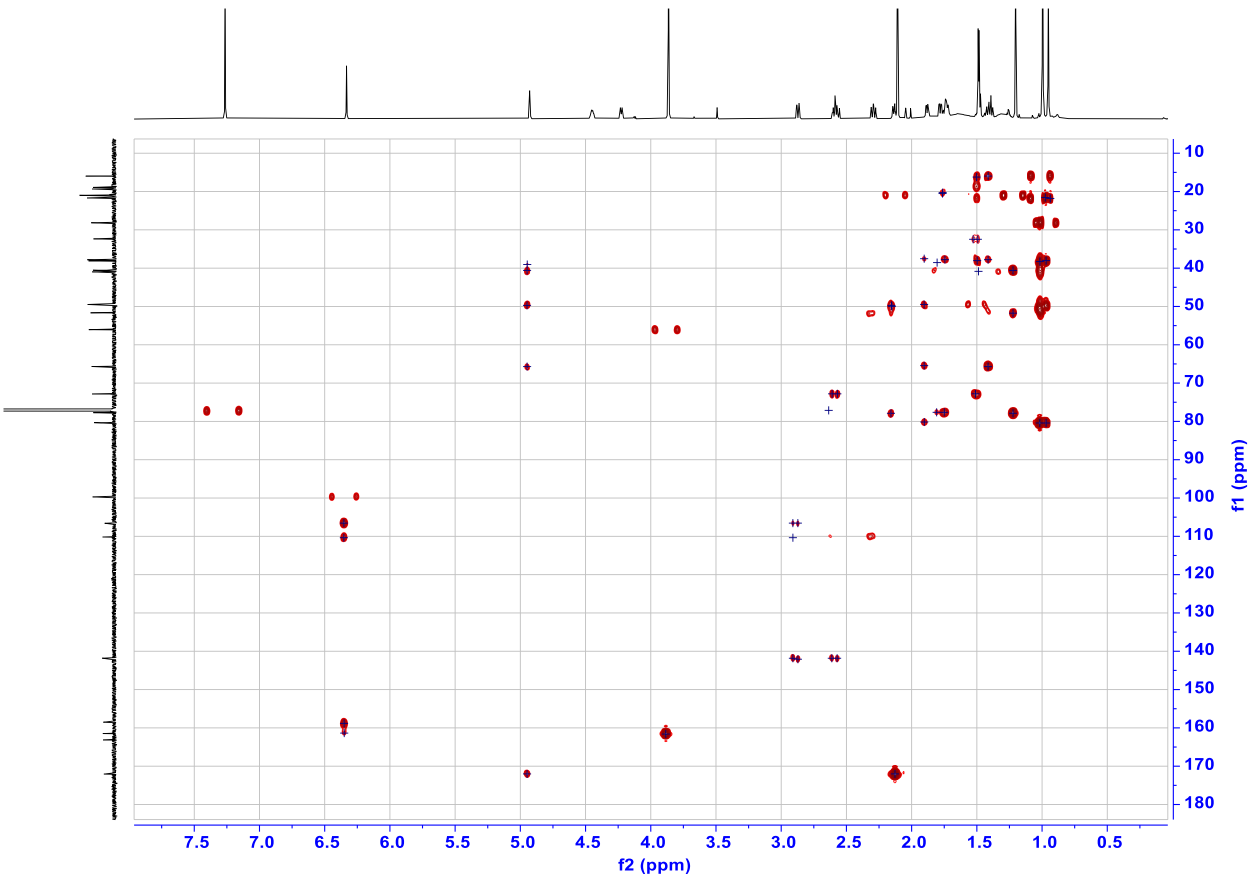

Figure S14. HMBC spectrum of **2**

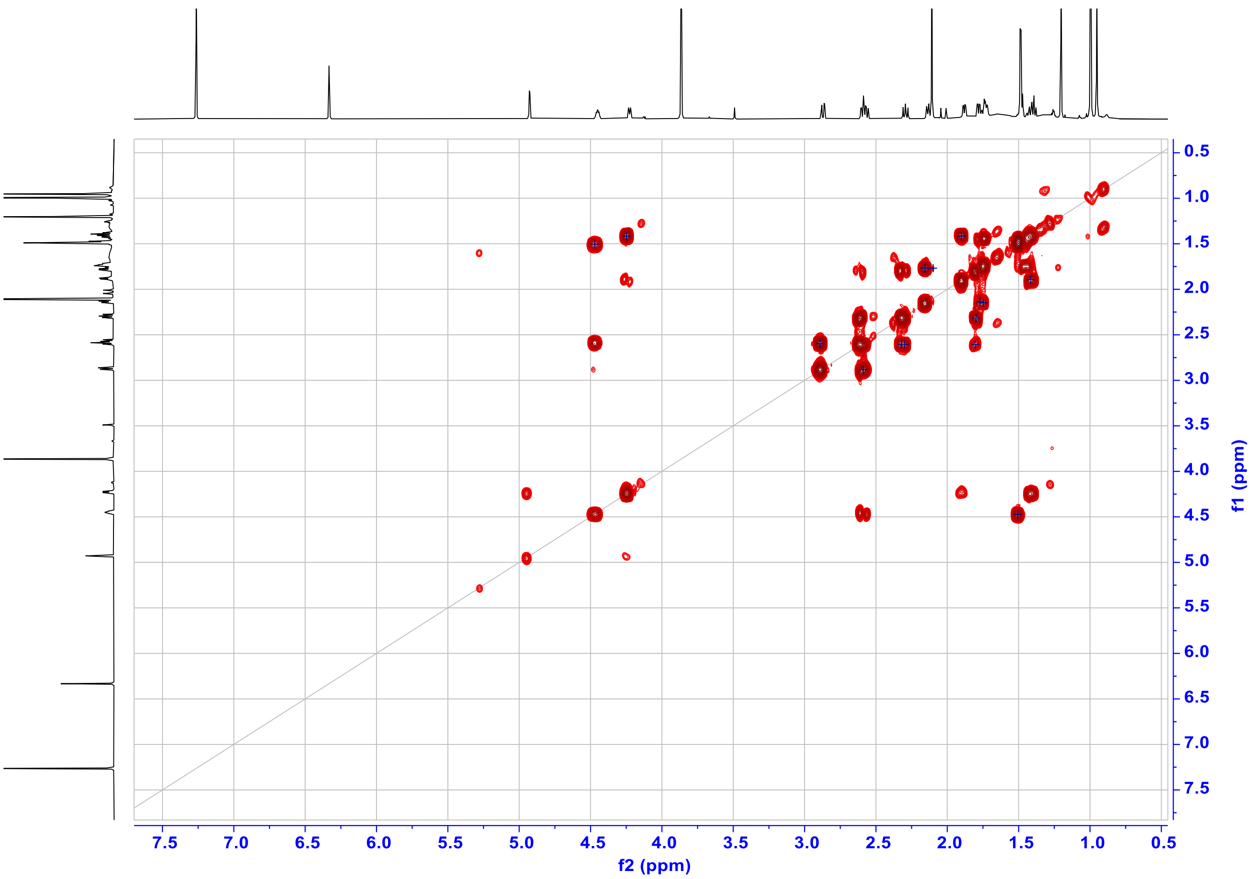

Figure S15. COSY spectrum of **2**

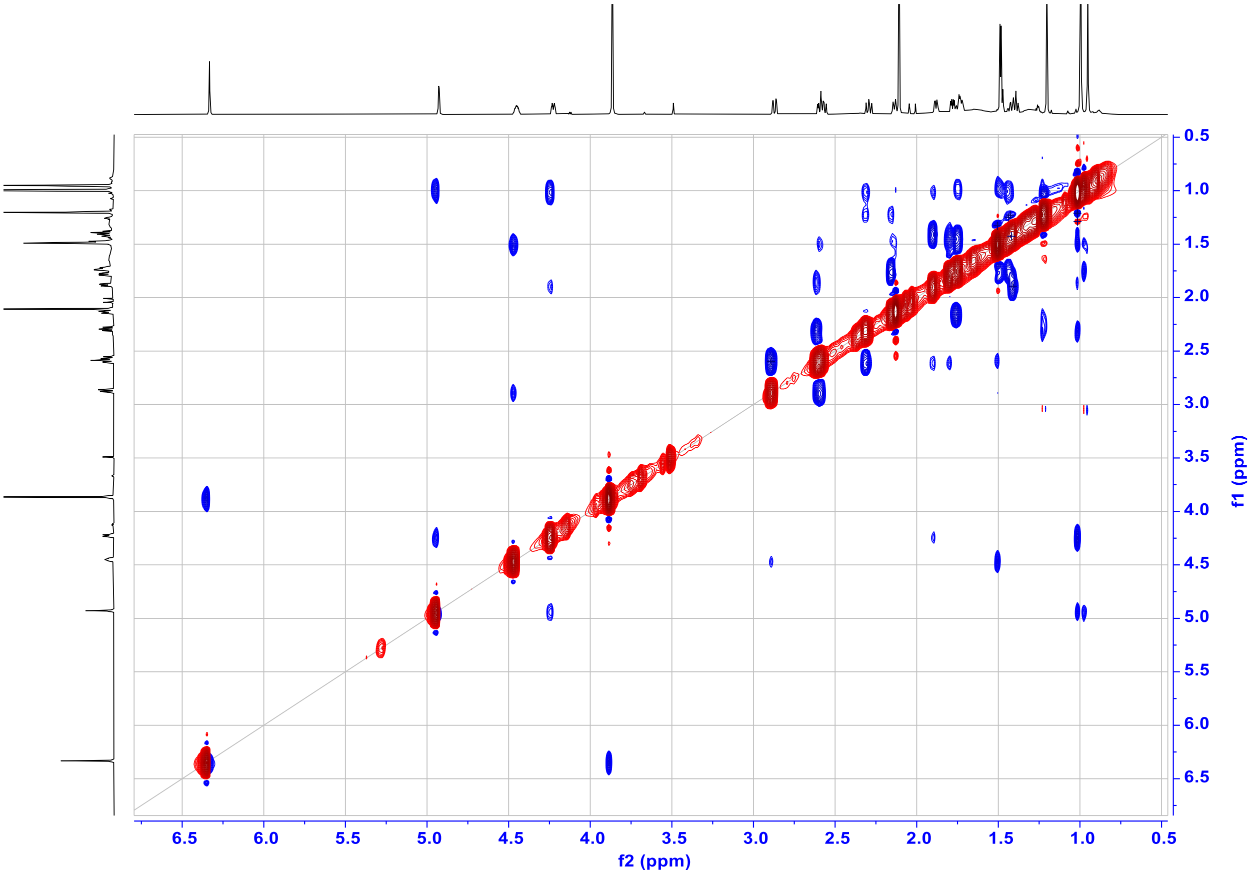

Figure S16. NOESY spectrum of **2**

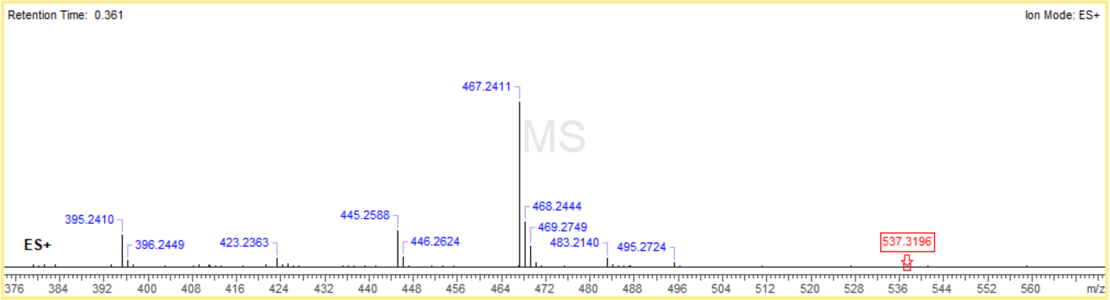

Figure S17. MS spectrum of **3**

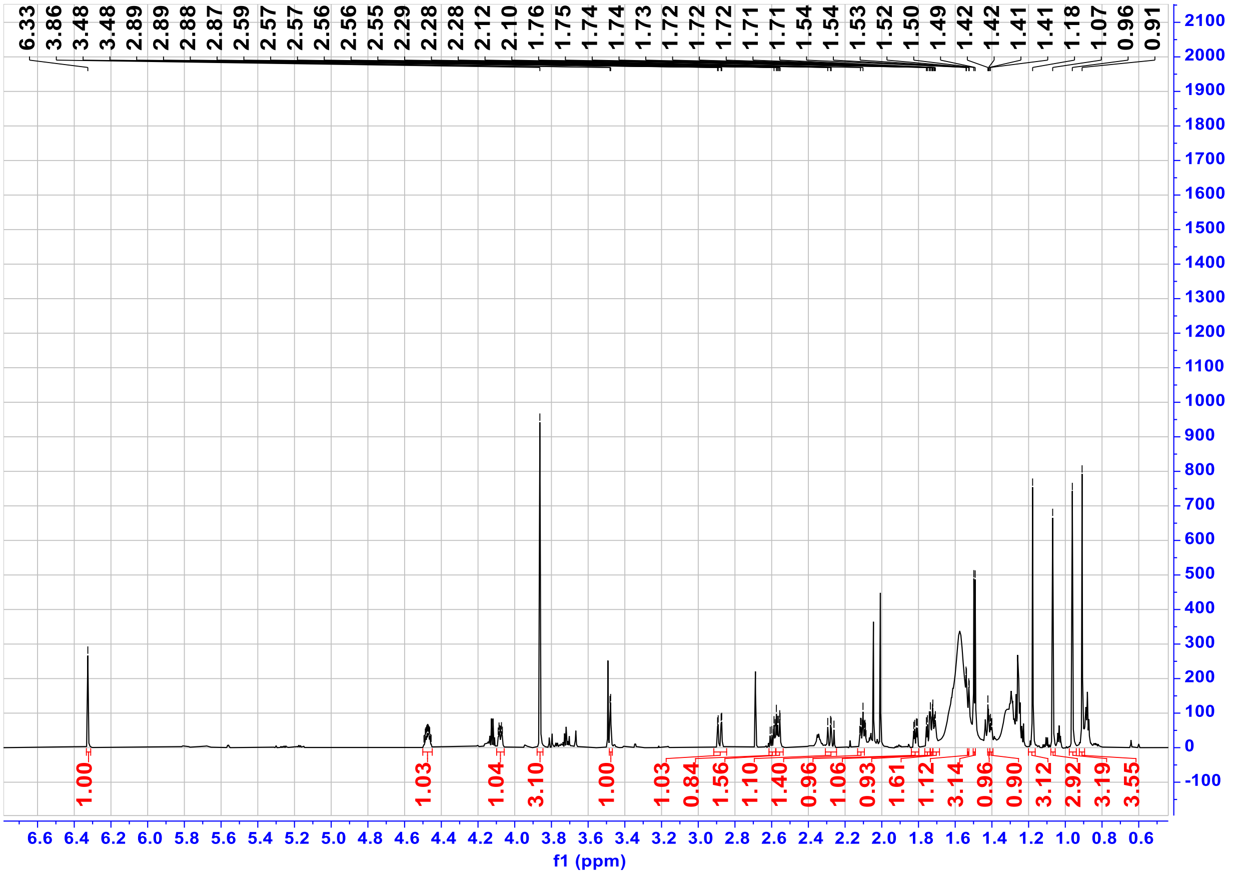

Figure S18. ^{1}H NMR spectrum of **3**

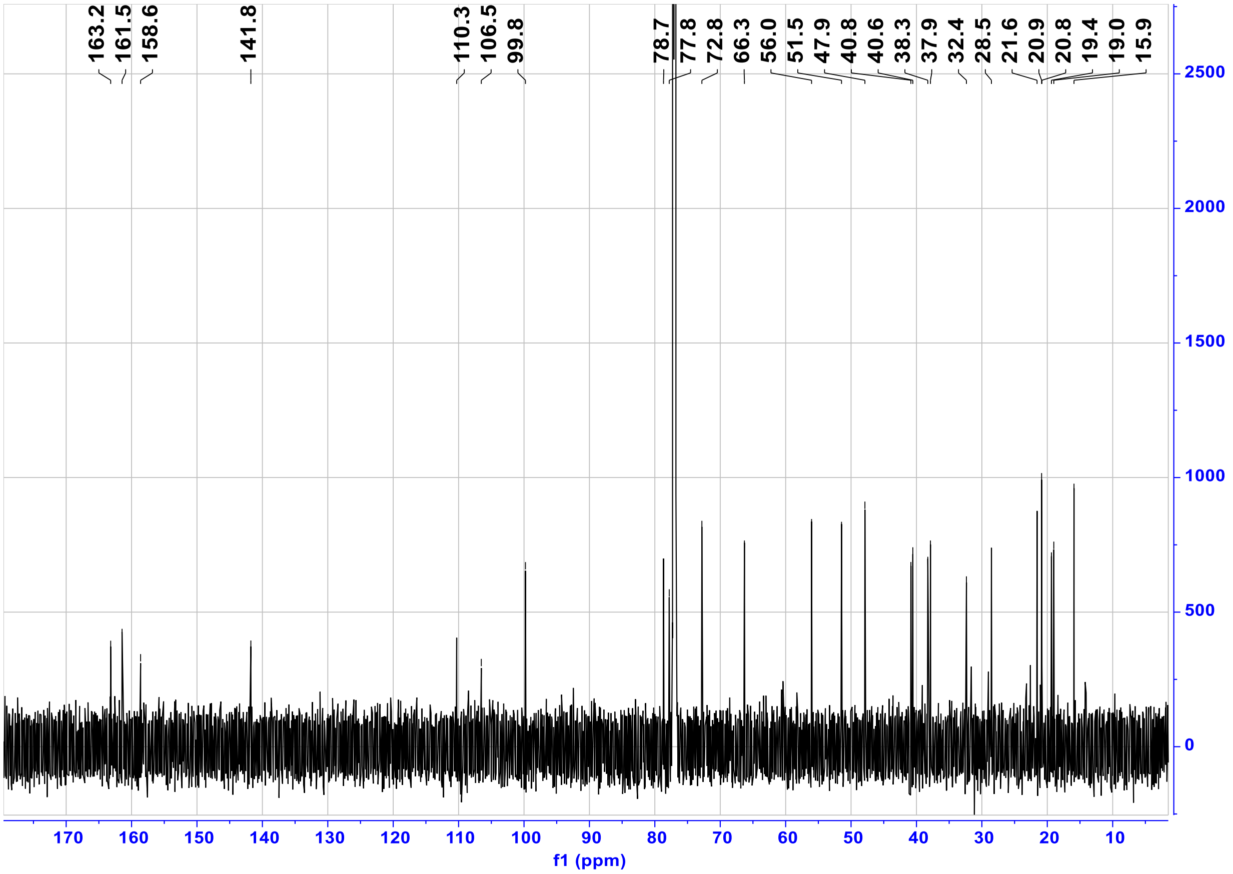

Figure S19. ^{13}C NMR spectrum of **3**

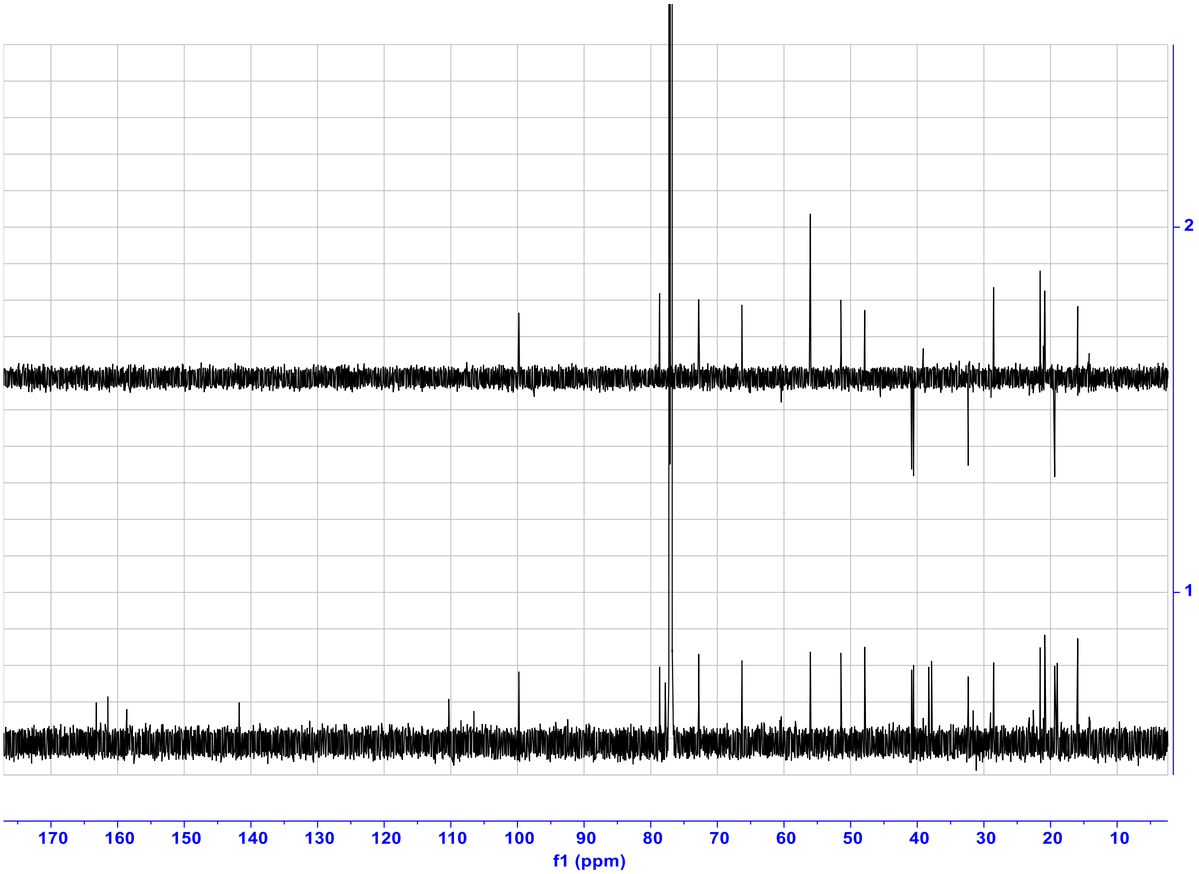

Figure S20. DEPT spectrum of **3**

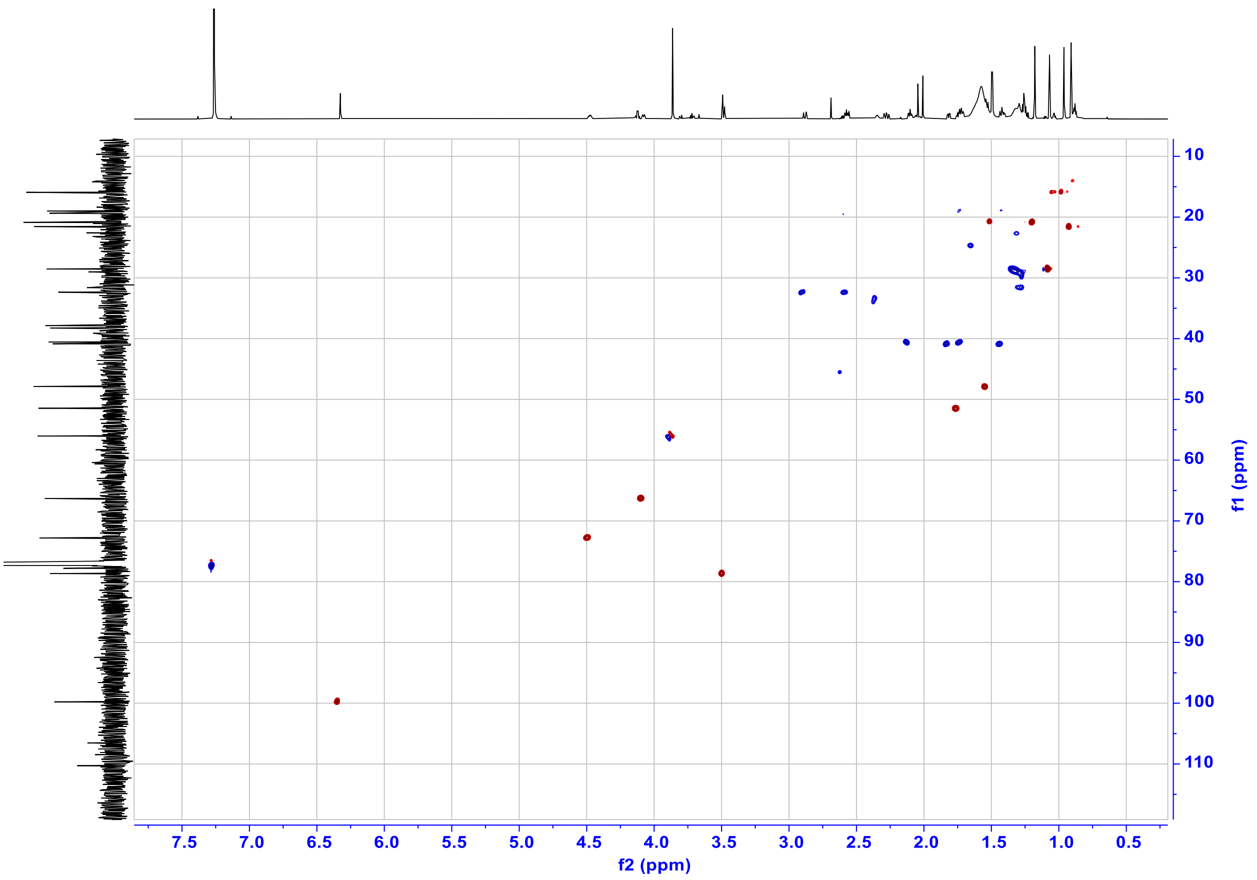

Figure S21. HSQC spectrum of **3**

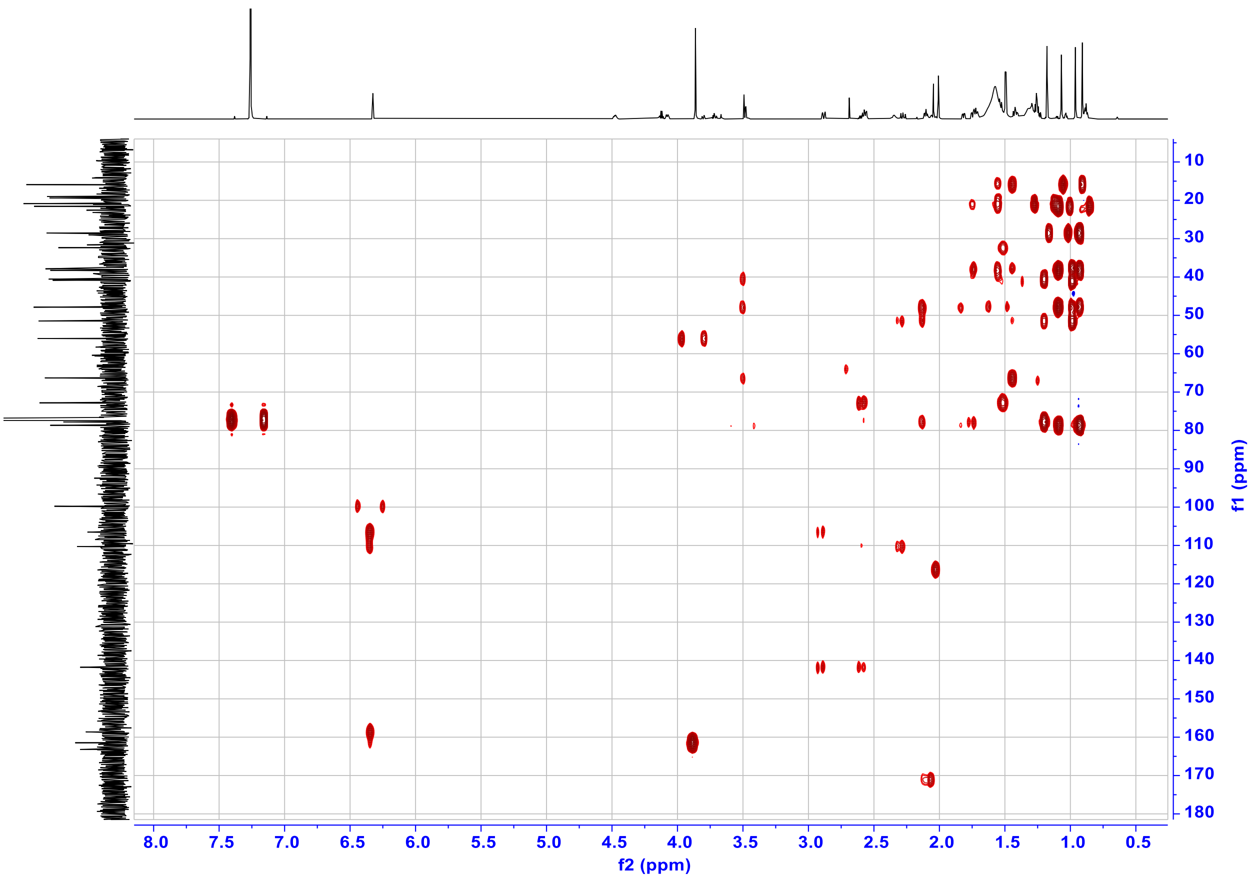

Figure S22. HMBC spectrum of **3**

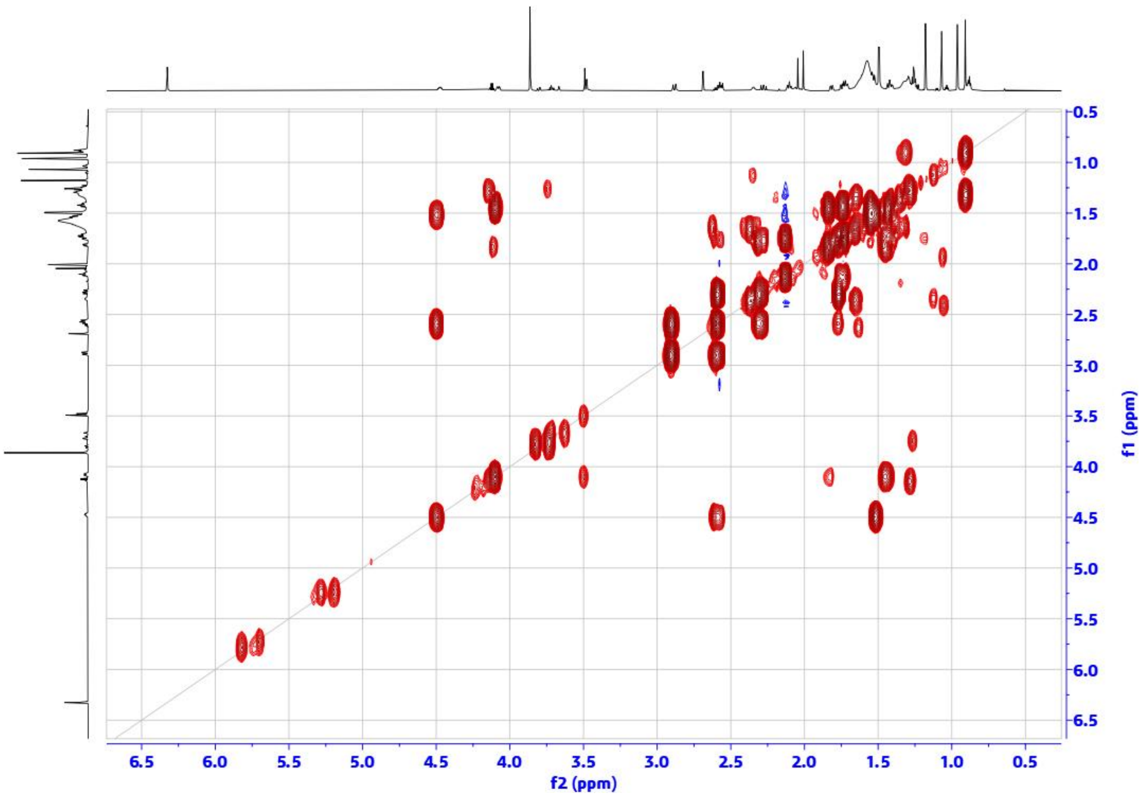

Figure S23. COSY spectrum of **3**

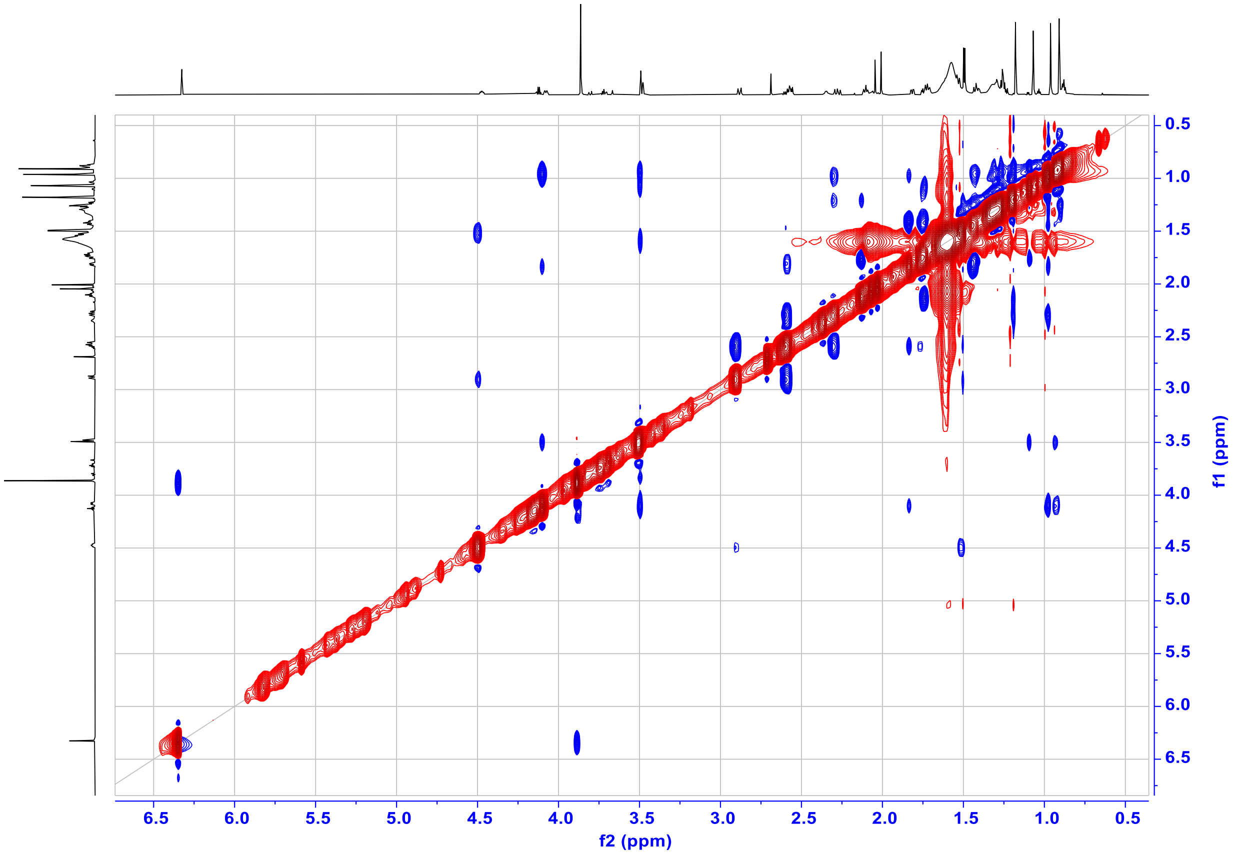

Figure S24. NOESY spectrum of **3**

Table S1. Experimental and calculated ^{13}C-NMR chemical shifts of 8'R-1

No.	δexptl.	8'R-1-δcalcd.
1	36.9	37.7
2	70.9	73.5
3	76.5	76.1
4	38.5	42.8
5	47.9	48.7
6	18.9	21.0
7	40.4	40.5
8	77.9	77.6
9	51.2	52.6
10	38.0	40.2
11	19.4	23.2
12	21.0	21.5
13	28.4	29.1
14	21.7	20.6
15	15.8	17.6
16	170.4	168.1
17	21.4	22.0
1'	110.7	112.4
2'	139.1	138.6
3'	101.7	101.5
4'	162.2	159.1
5'	103.4	99.7
6'	160.4	157.7
7‘	31.4	33.0
8'	74.7	74.8
9'	20.9	21.1
10'	170.1	166.5

Table S2. Experimental and calculated ^{1}H-NMR chemical shifts of 8'R-1

No.	δexptl.	8'R-1-δcalcd.
1a	1.8	1.59
1b	1.58	1.23
2	5.25	5.12
3	3.53	3.45
5	1.64	1.74
6a	1.72	1.6
6b	1.4	1.44
7a	2.09	1.95
7b	1.72	1.52
9	1.76	1.49
11a	2.48	2.28
11b	2.25	2.16
12	1.17	0.99
13	1.07	0.93
14	0.95	0.85
15	1.01	0.96
17	2.11	1.95
5'	6.28	6.37

7'a	2.95	2.79
7'b	2.61	2.5
8'	4.63	4.54
9'	1.55	1.22

Table S3. Conformational analysis of the B3LYP/6-31G(d) optimized conformers of 8'R-1 in the gas phase (T=298.15 K)

Conformer	E^a (Hartree)	C^b (Hartree)	G^c (kcal/mol)	ΔG^d (kcal/mol)	Population[e]
8'R-1-1	-1577.049219	0.530345	-989265.593272	0.0	40.06%
8'R-1-2	-1577.048494	0.529699	-989265.544019	0.049252	36.86%
8'R-1-3	-1577.047647	0.529636	-989265.051764	0.541507	16.05%
8'R-1-4	-1577.046857	0.529627	-989264.561994	1.031277	7.02%

[a]Electronic energy obtained at M062X/6-311+G(2d,p) level of theory; [b]Thermal correction to Gibbs free energy obtained at B3LYP/6-31G(d) level of theory; [c]Gibbs free energy (E + C); [d]The relative Gibbs free energy; [e]The Boltzmann distribution of each conformer.

Table S4. Atomic coordinates (Å) of 8'R-1-1 obtained at the B3LYP/6-31G(d) level of theory in the gas phase.

C	-2.136713	0.870693	-0.072247	H	-2.527133	-1.496531	-1.256705
C	-3.617733	1.221491	0.003601	H	3.995447	2.670437	-1.133564
C	-4.496622	0.207207	-0.722226	H	3.327255	1.824751	1.723619
C	-4.308954	-1.224657	-0.168616	H	2.175528	2.239129	0.460022
C	-2.786699	-1.564284	-0.191511	H	3.597223	-3.315998	-1.018746
C	-2.443250	-3.000109	0.236367	H	0.674756	0.219123	1.593226
C	-1.009974	-3.356100	-0.170893	H	0.534082	1.023012	0.048682
C	0.025244	-2.376513	0.385524	H	-2.578371	-3.133461	1.316243
C	-0.384507	-0.923584	0.064683	H	-3.122065	-3.711096	-0.242677
C	-1.834845	-0.533495	0.503823	H	-0.751730	-4.374588	0.141371
C	0.716830	0.046956	0.509773	H	-0.931732	-3.328833	-1.264192
C	0.353595	-2.674810	1.851917	H	-1.572382	1.638856	0.464356
C	-5.070809	-2.185766	-1.106050	H	-1.839910	0.905869	-1.126464
C	-4.969479	-1.326092	1.219520	H	-3.944044	1.346775	1.036159
C	-1.982009	-0.473708	2.044794	H	-5.550839	0.499598	-0.586256
C	-3.618972	3.619488	0.030240	H	6.489746	-1.278870	-0.856459
C	-3.893809	4.842051	-0.810639	H	4.545630	4.035903	1.559763
C	2.090062	-0.451470	0.116222	H	5.112695	4.605333	-0.023266
C	3.217348	0.367400	0.146220	H	3.372325	4.589414	0.342144
C	4.485113	-0.108852	-0.252655	H	-1.052096	-0.149000	2.518002
C	4.625859	-1.459677	-0.674335	H	-2.744109	0.245420	2.350510
C	3.502029	-2.281627	-0.711222	H	-2.252744	-1.435120	2.484367
C	2.262053	-1.788199	-0.313784	H	0.810981	-3.667065	1.907298
C	3.158792	1.793141	0.636912	H	1.062824	-1.953879	2.267323
C	4.212705	2.644442	-0.056734	H	-0.540025	-2.674434	2.476997
C	4.317630	4.056411	0.489067	H	-4.191047	1.132241	-2.393287
C	5.675122	0.739451	-0.184549	H	-4.585480	-2.265956	-2.081308
O	-3.852646	2.487792	-0.679136	H	-5.150170	-3.184917	-0.667120
O	5.809465	-1.981635	-1.027137	H	-6.090164	-1.818455	-1.270828
O	-4.176326	0.203147	-2.111740	H	-4.610041	-0.584940	1.934490
O	6.820038	0.319842	-0.353005	H	-6.053418	-1.193583	1.124335
O	1.231547	-2.668709	-0.379176	H	-4.799690	-2.315851	1.654874
O	-3.240120	3.624968	1.180144	H	-3.742201	5.738454	-0.209039
O	5.525909	2.052700	0.096166	H	-3.222283	4.858555	-1.675602

H	-0.398604	-0.889731	-1.033775	H	-4.919193	4.815854	-1.192652

Table S5. Atomic coordinates (Å) of 8'R-1-2 obtained at the B3LYP/6-31G(d) level of theory in the gas phase.

C	-2.179935	0.880776	-0.146369	H	-2.522105	-1.566214	-1.181229
C	-3.668830	1.190885	-0.052510	H	4.356525	3.644089	0.847922
C	-4.532215	0.111212	-0.697938	H	2.277353	2.192933	1.019314
C	-4.288096	-1.280629	-0.069636	H	2.788376	2.589236	-0.618041
C	-2.756881	-1.576150	-0.108156	H	3.628726	-3.154851	-1.120119
C	-2.360173	-2.971044	0.399710	H	0.689453	0.410386	1.476899
C	-0.925735	-3.310852	-0.017637	H	0.489212	1.120221	-0.109218
C	0.093242	-2.268313	0.448530	H	-2.468830	-3.040660	1.488581
C	-0.371130	-0.850553	0.049047	H	-3.026826	-3.730180	-0.018560
C	-1.821967	-0.477761	0.501444	H	-0.630112	-4.299448	0.352274
C	0.709087	0.178513	0.403519	H	-0.872656	-3.351486	-1.112085
C	0.468930	-2.462851	1.920849	H	-1.626641	1.694618	0.331330
C	-5.039618	-2.315533	-0.934158	H	-1.909031	0.863809	-1.207858
C	-4.915134	-1.323229	1.337153	H	-3.974114	1.364601	0.979483
C	-1.936697	-0.332008	2.039879	H	-5.591618	0.379652	-0.552934
C	-3.751794	3.584872	-0.154879	H	6.383642	-0.947452	-1.437014
C	-4.082244	4.750080	-1.054892	H	3.980423	2.369854	2.945976
C	2.084022	-0.305067	-0.001567	H	4.747456	0.934034	2.236774
C	3.177809	0.553255	-0.069078	H	5.708110	2.399550	2.529136
C	4.439849	0.106302	-0.512944	H	-1.006684	0.044936	2.472650
C	4.597775	-1.244998	-0.926204	H	-2.712497	0.382634	2.320814
C	3.515668	-2.117429	-0.829240	H	-2.170719	-1.273286	2.539745
C	2.284737	-1.655800	-0.369120	H	0.949633	-3.439748	2.027618
C	3.063773	2.016972	0.279248	H	1.173890	-1.701766	2.266328
C	4.383345	2.551599	0.829374	H	-0.406561	-2.438543	2.570508
C	4.726999	2.027995	2.220657	H	-4.291363	0.948065	-2.425403
C	5.583599	1.014674	-0.594994	H	-4.576404	-2.431532	-1.916543
O	-3.958947	2.409116	-0.797922	H	-5.073573	-3.291893	-0.441231
O	5.760547	-1.719526	-1.396107	H	-6.074763	-1.991235	-1.090707
O	-4.243958	0.037218	-2.092504	H	-6.004330	-1.227394	1.260024
O	6.652585	0.711632	-1.125294	H	-4.706446	-2.281459	1.823286
O	1.288026	-2.575374	-0.328516	H	-4.562319	-0.533136	2.001108
O	-3.353270	3.665110	0.985582	H	-5.109414	4.664736	-1.423140
O	5.473320	2.259989	-0.082447	H	-3.959074	5.682037	-0.502901
H	-0.413421	-0.886392	-1.048443	H	-3.420028	4.746460	-1.927188

Table S6. Atomic coordinates (Å) of 8'R-1-3 obtained at the B3LYP/6-31G(d) level of theory in the gas phase.

C	-2.041179	0.837331	-0.024824	H	-2.357108	-1.514415	-1.261982
C	-3.529816	1.136027	0.068820	H	4.020155	2.857358	-1.066215
C	-4.378868	0.109884	-0.681731	H	3.395040	1.931502	1.775833
C	-4.144912	-1.328494	-0.166073	H	2.223589	2.332040	0.525288
C	-2.612106	-1.615557	-0.198547	H	3.823913	-3.137725	-1.080305
C	-2.219934	-3.047482	0.196455	H	0.795579	0.237054	1.621497
C	-0.777239	-3.349464	-0.221453	H	0.625094	1.071568	0.095286
C	0.227395	-2.349710	0.354900	H	-2.346727	-3.209066	1.273380
C	-0.231060	-0.903662	0.069629	H	-2.877650	-3.768971	-0.295829
C	-1.692923	-0.569978	0.518975	H	-0.486012	-4.365779	0.068134
C	0.839779	0.091744	0.534058	H	-0.702046	-3.296040	-1.314000

C	0.571944	-2.672255	1.812662	H	-1.493584	1.613234	0.521194
C	-4.873684	-2.289565	-1.129804	H	-1.745849	0.905378	-1.077820
C	-4.802481	-1.489946	1.217719	H	-3.872130	1.221420	1.099264
C	-1.839723	-0.551545	2.059950	H	-5.440320	0.366535	-0.537232
C	-4.714859	3.222808	-0.044517	H	6.647719	-1.009509	-0.882402
C	-4.825509	4.510503	-0.823365	H	4.535967	4.186300	1.652115
C	2.227415	-0.351558	0.125123	H	5.077572	4.805863	0.079092
C	3.326607	0.503980	0.168712	H	3.340281	4.724948	0.449243
C	4.607768	0.079386	-0.245195	H	-2.640113	0.117583	2.380992
C	4.791587	-1.256408	-0.697116	H	-2.061948	-1.534314	2.478495
C	3.695586	-2.114457	-0.748679	H	-0.926164	-0.191679	2.540348
C	2.441493	-1.671615	-0.336357	H	1.067210	-3.647333	1.840827
C	3.222278	1.916472	0.689401	H	1.255009	-1.936052	2.244985
C	4.243426	2.817082	0.008919	H	-0.318286	-2.723561	2.440869
C	4.303212	4.220559	0.582760	H	-4.041420	1.092025	-2.315382
C	5.768757	0.965819	-0.164038	H	-4.915268	-3.303678	-0.720681
O	-3.737365	2.433568	-0.565548	H	-5.906285	-1.955056	-1.281093
O	5.990358	-1.730603	-1.065544	H	-4.388780	-2.323042	-2.108073
O	-4.055884	0.152283	-2.071044	H	-4.595922	-2.484069	1.626869
O	6.926323	0.589045	-0.345726	H	-4.474404	-0.753727	1.952405
O	1.439912	-2.583071	-0.420155	H	-5.890007	-1.393588	1.123647
O	-5.392924	2.916813	0.909648	H	-5.146784	4.298207	-1.848882
O	5.576282	2.267285	0.144975	H	-5.551766	5.165293	-0.341480
H	-0.249169	-0.844765	-1.027709	H	-3.850715	5.004105	-0.881841

Table S7. Atomic coordinates (Å) of 8'R-1-4 obtained at the B3LYP/6-31G(d) level of theory in the gas phase.

C	-2.083833	0.864485	-0.102874	H	-2.361544	-1.569605	-1.187077
C	-3.577599	1.129633	0.006708	H	4.384259	3.778757	0.938682
C	-4.414011	0.038283	-0.661474	H	2.347727	2.265515	1.090612
C	-4.131389	-1.359571	-0.065667	H	2.832211	2.718175	-0.540522
C	-2.592379	-1.609438	-0.114005	H	3.826775	-2.987114	-1.192662
C	-2.154447	-3.002169	0.363019	H	0.807212	0.431925	1.507443
C	-0.713384	-3.294929	-0.066724	H	0.580756	1.174931	-0.060886
C	0.278727	-2.235316	0.417814	H	-2.256265	-3.097766	1.450479
C	-0.226487	-0.821605	0.053278	H	-2.802631	-3.769932	-0.068044
C	-1.686620	-0.497693	0.515943	H	-0.389590	-4.282712	0.281113
C	0.827646	0.227340	0.428372	H	-0.663596	-3.311169	-1.161932
C	0.669600	-2.453298	1.883099	H	-1.545622	1.684726	0.384340
C	-4.853762	-2.395768	-0.953338	H	-1.812082	0.877142	-1.164183
C	-4.756453	-1.451845	1.339666	H	-3.899688	1.267507	1.037998
C	-1.801158	-0.389201	2.056320	H	-5.478716	0.276385	-0.509041
C	-4.818941	3.175949	-0.200134	H	6.518388	-0.698469	-1.473444
C	-4.983748	4.409772	-1.052959	H	4.061421	2.441716	3.006766
C	2.213233	-0.208734	0.005427	H	4.861870	1.046429	2.254774
C	3.283021	0.680275	-0.047088	H	5.783891	2.530312	2.577064
C	4.553574	0.279056	-0.509887	H	-0.889436	0.026904	2.492962
C	4.745043	-1.056858	-0.957683	H	-2.615515	0.273679	2.354307
C	3.687725	-1.960667	-0.875377	H	-1.983222	-1.350986	2.537867
C	2.447916	-1.544071	-0.396671	H	1.178313	-3.418452	1.963585
C	3.131777	2.131083	0.339267	H	1.355874	-1.681397	2.242067
C	4.440981	2.688204	0.892168	H	-0.201193	-2.469861	2.539590

C	4.810873	2.139403	2.266962	H	-4.137814	0.927574	-2.358869
C	5.671252	1.220397	-0.578159	H	-4.857925	-3.384350	-0.483980
O	-3.834657	2.380513	-0.698445	H	-5.898335	-2.098394	-1.099791
O	5.916646	-1.487824	-1.446867	H	-4.389893	-2.474549	-1.939210
O	-4.121611	0.004472	-2.057814	H	-4.435361	-0.661258	2.018810
O	6.743982	0.960028	-1.122988	H	-5.847916	-1.392047	1.263174
O	1.476557	-2.490667	-0.372945	H	-4.512946	-2.411358	1.806918
O	-5.464344	2.912419	0.788783	H	-5.704464	5.080949	-0.585467
O	5.530264	2.449942	-0.035288	H	-4.021902	4.915398	-1.180678
H	-0.270785	-0.833183	-1.044645	H	-5.339892	4.127205	-2.049653

Table S8. Experimental and calculated ^{13}C-NMR chemical shifts of 8’S-1

No.	δexptl.	8S-1-δcalcd.
1	36.9	37.7
2	70.9	73.3
3	76.5	76.0
4	38.5	42.9
5	47.9	49.1
6	18.9	20.7
7	40.4	40.7
8	77.9	77.4
9	51.2	52.0
10	38.0	40.3
11	19.4	23.1
12	21.0	21.5
13	28.4	29.0
14	21.7	20.4
15	15.8	17.8
16	170.4	168.1
17	21.4	22.1
1'	110.7	113.0
2'	139.1	139.1
3'	101.7	102.0
4'	162.2	159.2
5'	103.4	99.1
6'	160.4	157.3
7‘	31.4	34.8
8'	74.7	74.6
9'	20.9	21.6
10'	170.1	166.4

Table S9. Experimental and calculated ^{1}H-NMR chemical shifts of 8’S-1

No.	δexptl.	8S-1-δcalcd.
1a	1.8	1.64
1b	1.58	1.22
2	5.25	5.14
3	3.53	3.46
5	1.64	1.73
6a	1.72	1.61
6b	1.4	1.44
7a	2.09	1.96

7b	1.72	1.53
9	1.76	1.52
11a	2.48	2.34
11b	2.25	2.12
12	1.17	1.02
13	1.07	0.95
14	0.95	0.86
15	1.01	0.97
17	2.11	1.96
5'	6.28	6.36
7'a	2.95	2.57
7'b	2.61	2.53
8'	4.63	4.42
9'	1.55	1.34

Table S10. Conformational analysis of the B3LYP/6-31G(d) optimized conformers of 8’S-1 in the gas phase (T=298.15 K)

Conformer	E[a] (Hartree)	C[b] (Hartree)	G[c] (kcal/mol)	ΔG[d] (kcal/mol)	Population[e]
8S-1-1	-1577.04992	0.529763	-989266.398794	0.0	76.30%
8S-1-2	-1577.048304	0.529386	-989265.620963	0.77783	20.51%
8S-1-3	-1577.048033	0.53087	-989264.519739	1.879055	3.19%

[a]Electronic energy obtained at M062X/6-311+G(2d,p) level of theory; [b]Thermal correction to Gibbs free energy obtained at B3LYP/6-31G(d) level of theory; [c]Gibbs free energy (E + C); [d]The relative Gibbs free energy; [e]The Boltzmann distribution of each conformer.

Table S11. Atomic coordinates (Å) of 8’S-1-1 obtained at the B3LYP/6-31G(d) level of theory in the gas phase.

C	-2.136865	0.879585	-0.136635	H	-2.543178	-1.552675	-1.181194
C	-3.615819	1.240385	-0.071671	H	4.690357	1.772420	1.874515
C	-4.503057	0.189922	-0.732747	H	2.359315	2.021404	1.156676
C	-4.317749	-1.208855	-0.100073	H	2.862925	2.439113	-0.479758
C	-2.796945	-1.556160	-0.112367	H	3.548237	-3.342486	-1.018722
C	-2.455837	-2.965196	0.398273	H	0.686301	0.308060	1.541951
C	-1.026184	-3.349838	0.004366	H	0.535469	1.033136	-0.042574
C	0.017670	-2.342937	0.493135	H	-2.585848	-3.034298	1.484929
C	-0.391492	-0.909435	0.089203	H	-3.139515	-3.700894	-0.034320
C	-1.836982	-0.490562	0.516225	H	-0.769348	-4.348920	0.375038
C	0.715995	0.081587	0.467576	H	-0.955308	-3.387956	-1.089197
C	0.356542	-2.553259	1.972181	H	-1.565850	1.674070	0.352498
C	-5.089037	-2.218177	-0.977241	H	-1.845646	0.853530	-1.192505
C	-4.969442	-1.229853	1.295938	H	-3.934707	1.425961	0.954142
C	-1.975189	-0.342245	2.052575	H	-5.555347	0.493703	-0.607437
C	-3.610451	3.635645	-0.178640	H	6.371895	-1.220783	-1.310405
C	-3.879459	4.810249	-1.086894	H	3.785627	4.102439	1.947228
C	2.080316	-0.445848	0.082229	H	5.520526	4.101603	1.556660
C	3.202910	0.376407	0.035632	H	4.324234	4.364020	0.271593
C	4.456432	-0.110623	-0.390853	H	-2.260393	-1.272861	2.545875
C	4.575260	-1.465322	-0.807744	H	-1.038627	-0.006601	2.504641
C	3.465176	-2.302663	-0.726303	H	-2.724697	0.405153	2.319884
C	2.242674	-1.801573	-0.284279	H	1.079370	-1.817192	2.334413
C	3.130952	1.838437	0.402356	H	-0.530975	-2.500625	2.603640
C	4.468737	2.318832	0.947327	H	0.801495	-3.546157	2.086100

C	4.528797	3.814453	1.196235	H	-4.200967	1.016646	-2.455480
C	5.621683	0.767857	-0.486149	H	-6.109240	-1.858256	-1.152832
O	-3.849458	2.466903	-0.823423	H	-4.612353	-2.351992	-1.950865
O	5.727286	-1.974545	-1.268102	H	-5.165933	-3.191872	-0.483954
O	-4.191405	0.104976	-2.121596	H	-4.798728	-2.193449	1.786295
O	6.681297	0.437507	-1.018244	H	-4.603736	-0.450329	1.965425
O	1.216747	-2.688758	-0.260715	H	-6.053805	-1.100646	1.200852
O	-3.230310	3.703756	0.968872	H	-4.899345	4.759264	-1.480719
O	5.536421	2.020413	0.014643	H	-3.738251	5.738203	-0.532464
H	-0.414357	-0.940744	-1.009058	H	-3.196023	4.783117	-1.942278

Table S12. Atomic coordinates (Å) of 8'S-1-2 obtained at the B3LYP/6-31G(d) level of theory in the gas phase.

C	-2.044083	0.850379	-0.089732	H	-2.372476	-1.575210	-1.180339
C	-3.531463	1.157713	-0.007786	H	4.767832	1.922391	1.908786
C	-4.386010	0.090162	-0.691148	H	2.424900	2.107699	1.212494
C	-4.154474	-1.314608	-0.089789	H	2.901598	2.575797	-0.418601
C	-2.622706	-1.608271	-0.111331	H	3.770696	-3.167242	-1.085907
C	-2.232886	-3.013720	0.371775	H	0.808020	0.331671	1.569922
C	-0.793614	-3.345577	-0.034494	H	0.627161	1.086726	0.001392
C	0.219646	-2.314637	0.467909	H	-2.356216	-3.107408	1.457236
C	-0.238198	-0.887074	0.095628	H	-2.894666	-3.762630	-0.071739
C	-1.696006	-0.522943	0.534515	H	-0.502965	-4.342334	0.316979
C	0.839221	0.130570	0.490367	H	-0.726578	-3.361850	-1.128776
C	0.578010	-2.543657	1.939689	H	-1.492019	1.655280	0.407618
C	-4.890468	-2.330607	-0.989575	H	-1.752872	0.855943	-1.145870
C	-4.806816	-1.387687	1.304110	H	-3.868885	1.306090	1.017097
C	-1.833662	-0.411501	2.072795	H	-5.446133	0.358688	-0.558901
C	-4.709684	3.238397	-0.239381	H	6.522583	-0.949885	-1.348560
C	-4.824435	4.474565	-1.097034	H	5.519004	4.284021	1.630158
C	2.217743	-0.344026	0.086775	H	4.303160	4.531540	0.360649
C	3.312809	0.514966	0.050994	H	3.788840	4.219159	2.035390
C	4.578517	0.078137	-0.393366	H	-2.045863	-1.368657	2.551332
C	4.738167	-1.262864	-0.839995	H	-0.920136	-0.016093	2.524758
C	3.656151	-2.137270	-0.770257	H	-2.637531	0.270056	2.356902
C	2.421060	-1.685524	-0.310631	H	1.279259	-1.791600	2.311436
C	3.196080	1.966066	0.448748	H	-0.304750	-2.534679	2.579805
C	4.521621	2.479300	0.994112	H	1.057080	-3.523061	2.029049
C	4.534205	3.970955	1.272378	H	-4.051749	0.969186	-2.383550
C	5.713843	0.995912	-0.478032	H	-4.934225	-3.317218	-0.518113
O	-3.739396	2.414594	-0.718622	H	-5.922315	-2.001837	-1.157490
O	5.902764	-1.724655	-1.318025	H	-4.409654	-2.426555	-1.965758
O	-4.068146	0.046384	-2.081609	H	-5.894701	-1.298045	1.207897
O	6.779911	0.711400	-1.023199	H	-4.598942	-2.352915	1.776774
O	1.423841	-2.604877	-0.301252	H	-4.476892	-0.605446	1.988701
O	-5.380089	2.994972	0.738006	H	-3.846327	4.951677	-1.209982
O	5.591123	2.235034	0.048493	H	-5.172330	4.199974	-2.098879
H	-0.264388	-0.896453	-1.002875	H	-5.532992	5.167465	-0.642839

Table S13. Atomic coordinates (Å) of 8'S-1-3 obtained at the B3LYP/6-31G(d) level of theory in the gas phase.

C	-2.139199	0.861357	-0.001165	H	-2.454705	-1.435616	-1.330222
C	-3.628589	1.176933	0.068442	H	4.228088	3.687670	0.900409

C	-4.473721	0.189341	-0.730966	H	3.305168	1.775953	2.010067
C	-4.262051	-1.269584	-0.262926	H	2.128928	2.267015	0.800133
C	-2.732169	-1.573997	-0.276603	H	3.708716	-3.134734	-1.063964
C	-2.364124	-3.024767	0.070810	H	0.644046	0.175476	1.686986
C	-0.915670	-3.325742	-0.327470	H	0.527480	1.062365	0.186268
C	0.085419	-2.360737	0.310962	H	-2.514330	-3.225573	1.138080
C	-0.350438	-0.900008	0.069990	H	-3.018186	-3.720226	-0.462287
C	-1.818192	-0.567259	0.499219	H	-0.641556	-4.355929	-0.072303
C	0.718300	0.065745	0.597357	H	-0.815349	-3.229635	-1.415070
C	0.388762	-2.742065	1.763528	H	-1.600020	1.608858	0.587936
C	-4.983630	-2.187172	-1.273261	H	-1.826074	0.962060	-1.046468
C	-4.945610	-1.474662	1.102349	H	-3.974630	1.236576	1.100500
C	-1.998778	-0.602726	2.037273	H	-5.536207	0.450080	-0.594635
C	-3.684203	3.568846	0.234335	H	6.546724	-1.041640	-0.726317
C	-3.974306	4.832161	-0.538353	H	4.649120	3.763192	-1.555492
C	2.111884	-0.380885	0.213085	H	3.746429	2.247531	-1.768201
C	3.220242	0.455229	0.327534	H	2.908064	3.706315	-1.201751
C	4.506522	0.033445	-0.071865	H	-2.271334	-1.591058	2.411112
C	4.686892	-1.282034	-0.579658	H	-1.082412	-0.301755	2.550763
C	3.583112	-2.124876	-0.692545	H	-2.773397	0.090864	2.369358
C	2.323476	-1.683865	-0.296287	H	0.869369	-3.724860	1.767897
C	3.126226	1.836320	0.927338	H	1.070905	-2.032337	2.239215
C	4.152422	2.776502	0.301502	H	-0.517939	-2.802023	2.366895
C	3.846256	3.143068	-1.147332	H	-4.162541	1.221375	-2.336957
C	5.668793	0.915062	0.044416	H	-4.479737	-2.193266	-2.242301
O	-3.879194	2.475908	-0.544195	H	-5.045441	-3.213883	-0.900000
O	5.888243	-1.752239	-0.944679	H	-6.008940	-1.834690	-1.433472
O	-4.132687	0.277143	-2.112715	H	-6.030667	-1.363279	0.993654
O	6.825617	0.538343	-0.144800	H	-4.757684	-2.485900	1.476664
O	1.313957	-2.578792	-0.443106	H	-4.619472	-0.772095	1.870195
O	-3.325972	3.514930	1.389568	H	-4.999614	4.813140	-0.921316
O	5.484208	2.208119	0.388702	H	-3.835433	5.696014	0.111915
H	-0.342567	-0.800000	-1.024576	H	-3.303922	4.904928	-1.401135

Functional	Solvent?		Basis Set		Type of Data	
mPW1PW91	PCM		6-31+G(d,p)		Unscaled Shifts	
	Isomer 1	Isomer 2	Isomer 3	Isomer 4	Isomer 5	Isomer 6
sDP4+ (H data)	97.84%	2.16%	–	–	–	–
sDP4+ (C data)	95.86%	4.14%	–	–	–	–
sDP4+ (all data)	99.90%	0.10%	–	–	–	–
uDP4+ (H data)	55.61%	44.39%	–	–	–	–
uDP4+ (C data)	81.54%	18.46%	–	–	–	–
uDP4+ (all data)	84.70%	15.30%	–	–	–	–
DP4+ (H data)	98.27%	1.73%	–	–	–	–
DP4+ (C data)	99.03%	0.97%	–	–	–	–
DP4+ (all data)	99.98%	0.02%	–	–	–	–

Figure S25. Result of DP4+ analysis (Isomer 1: 8'R-1; Isomer 2: 8'S-1)

Table S14. Key transitions, oscillator strengths, and rotatory strengths in the ECD spectrum of conformer 8'R-1-1 at the CAM-B3LYP/6-311G(d) level of theory in MeOH with IEFPCM solvent model.

Num[a]	*Transition*[b]	*CI-coeff*[c]	*ΔE (eV)*[d]	*λ (nm)*[e]	*f*[f]	R_{vel}[g]	R_{len}[h]

1	127->128	0.65522	4.4591	278.05	0.1544	10.6858	10.727
2	126->128	0.62937	4.9920	248.37	0.2755	-6.5486	-1.2189
3	121->128	0.47124	5.5405	223.78	0.0014	0.4656	1.0121
	122->128	-0.33622					
	123->128	0.23653					
4	126->128	-0.25277	5.8865	210.63	0.3417	-4.4731	-5.0703
	126->129	0.24949					
	127->129	0.58425					
5	121->130	0.2642	5.9789	207.37	0.0013	3.9272	2.9594
	122->130	0.5358					
	124->130	0.29797					
6	126->129	0.60759	6.2462	198.49	0.6191	24.6553	26.0958
	127->129	-0.2805					
7	119->128	0.49101	6.6305	186.99	0.0807	26.2805	26.5288
	124->128	-0.25614					
8	118->128	-0.27856	6.8606	180.72	0.0903	-5.9778	-7.4736
	119->128	0.4063					
	124->128	0.30457					
9	117->128	0.33348	7.0886	174.91	0.0514	-14.4246	-17.3807
	118->128	0.38297					
10	127->131	0.46617	7.3252	169.26	0.0348	4.2317	2.6419
	127->132	-0.26578					
11	125->128	0.36033	7.3805	167.99	0.0103	-7.3575	-7.8428
	127->131	0.26115					
12	111->128	-0.29309	7.4963	165.39	0.0129	-5.1525	-6.2995
	112->128	0.37037					
	124->128	0.24672					
13	127->135	0.49987	7.5426	164.38	0.0474	-12.9485	-12.2432
14	121->129	-0.3103	7.6879	161.27	0.0072	-10.3756	-10.5267
	122->129	0.27055					
	125->128	0.32721					
15	109->128	0.28593	7.7835	159.29	0.0176	5.0758	6.2969
	121->129	0.22383					
16	126->131	0.3775	7.8459	158.02	0.0611	-2.179	-3.1588
	126->135	-0.22363					
17	125->130	0.30279	7.8598	157.74	0.0564	-41.5001	-46.5901
	127->130	0.52087					
18	123->128	0.23215	7.9305	156.34	0.0307	-12.6873	-24.0451
	125->130	0.26079					
	127->130	-0.22987					
19	123->129	0.26788	7.9382	156.19	0.0137	-13.4618	-12.4008
20	123->128	0.27913	7.9507	155.94	0.0201	-23.5515	-20.0529
21	125->130	-0.25602	7.9663	155.64	0.0287	33.5551	38.8517
	125->131	0.23961					
	125->132	0.28132					
22	126->135	-0.24444	7.9828	155.31	0.0646	-9.0284	-12.1508
	127->133	0.34487					
	127->136	-0.2488					
23	126->135	-0.24872	8.0365	154.28	0.0074	12.1407	12.1363
	127->134	0.34359					
	127->136	0.30522					

	127->138	-0.27099					
24	108->128	0.2403	8.0733	153.57	0.0007	-7.0817	-7.4658
	115->128	-0.24733					
	120->128	-0.23162					
	124->128	0.28908					
25	126->131	0.29649	8.1106	152.87	0.0309	8.3256	9.5104
	126->135	0.2964					
	127->134	0.28716					
26	126->134	0.24606	8.1323	152.46	0.0178	1.9344	0.6809
	126->144	0.29493					
27	115->128	0.32107	8.2267	150.71	0.0131	7.7951	7.238
	120->128	-0.22707					
	122->128	0.35671					
28	117->129	0.25521	8.2414	150.44	0.0686	10.2522	10.9126
	119->129	0.47536					
29	123->130	-0.22921	8.3661	148.20	0.0142	-5.0986	-7.438
	125->130	-0.26798					
	126->130	0.52406					
30	120->128	0.4003	8.3777	147.99	0.0006	-0.7855	-1.2158
	121->128	0.37446					
	122->128	0.34947					
31	116->130	0.27751	8.3953	147.68	0.0286	24.8654	24.1109
	123->130	0.2563					
	125->130	0.26294					
	126->130	0.36533					
32	115->128	0.23332	8.4099	147.43	0.0039	-5.6414	-7.0042
	123->128	-0.23249					
33	127->137	0.31853	8.4212	147.23	0.0014	2.4774	-3.0966
	127->144	-0.23889					
34	120->129	-0.23979	8.4352	146.98	0.0017	10.4919	13.8197
	121->129	0.24176					
	124->129	-0.23054					
	125->129	0.2886					
35	127->145	0.43316	8.4855	146.11	0.0009	-0.1167	4.8082
	127->147	0.2722					
36	117->129	0.25795	8.5364	145.24	0.0037	-4.0944	-4.811
	118->129	0.36119					
	119->129	-0.30424					

[a]Number of the excited states; [b]Only transitions with contribution over 10.0% were listed; [c]Configuration-interaction coefficient; [d]Excitation energy; [e]Wavelength; [f]Oscillator strength; [g]Rotatory strength in velocity form (10^{-40} cgs); [h]Rotatory strength in length form (10^{-40} cgs).

Table S15. Key transitions, oscillator strengths, and rotatory strengths in the ECD spectrum of conformer 8'R-1-2 at the CAM-B3LYP/6-311G(d) level of theory in MeOH with IEFPCM solvent model.

Num[a]	***Transition***[b]	***CI-coeff***[c]	***ΔE (eV)***[d]	***λ (nm)***[e]	***f***[f]	R_{vel}[g]	R_{len}[h]
1	127->128	0.65496	4.4538	278.38	0.1520	-6.8767	-7.7353
2	126->128	0.63263	4.9821	248.86	0.2725	41.6409	43.3858
3	121->128	0.41794	5.5251	224.40	0.0008	0.8704	1.1353
	122->128	-0.2973					
	123->128	0.36122					
4	126->128	-0.24669	5.8849	210.68	0.3245	-11.5776	-12.6648

	126->129	0.25249					
	127->129	0.58606					
5	121->130	0.29467	5.9802	207.32	0.0012	4.0549	3.0419
	122->130	0.54069					
	124->130	0.30354					
6	126->129	0.60573	6.2328	198.92	0.6145	35.5886	40.0281
	127->129	-0.28589					
7	119->128	0.49822	6.6779	185.66	0.0895	19.1462	18.7657
	124->128	-0.23276					
8	114->128	-0.27102	6.8597	180.74	0.0607	-5.6241	-7.0901
	119->128	0.27119					
	124->128	0.30246					
9	118->128	0.56798	7.1157	174.24	0.0626	-21.1222	-12.2796
10	127->131	0.58995	7.3196	169.39	0.0044	11.2417	11.2861
	127->132	0.28487					
11	125->128	0.39853	7.3390	168.94	0.0122	-8.8976	-8.7537
12	112->128	-0.33314	7.4078	167.37	0.0090	3.9447	3.4552
	114->128	0.28463					
	124->128	0.33517					
13	127->135	0.53972	7.4928	165.47	0.0810	-9.4158	-8.6362
14	121->129	-0.27496	7.6836	161.36	0.0016	-2.3591	-2.3882
	122->129	0.24111					
	123->129	-0.25911					
	125->128	0.34609					
15	109->128	0.28228	7.7681	159.61	0.0008	4.9032	3.3897
16	125->130	0.26313	7.8472	158.00	0.0225	5.0076	1.3383
	127->130	0.5951					
17	126->131	0.40056	7.8612	157.72	0.0159	-16.6516	-15.9748
	127->132	-0.25283					
	127->134	-0.27071					
18	126->131	-0.22845	7.9166	156.61	0.1272	68.3254	69.6692
	126->135	0.3461					
19	125->130	0.29214	7.9315	156.32	0.0484	-81.2623	-85.2659
	126->135	0.22678					
20	123->128	0.23719	7.9361	156.23	0.0421	-27.1744	-32.926
	125->130	0.2608					
21	126->131	0.32276	7.9527	155.90	0.0410	-26.5652	-26.7939
22	125->132	0.2988	7.9644	155.67	0.0121	21.3211	24.5316
23	123->128	0.24008	7.9763	155.44	0.0253	1.0236	3.0597
	126->135	0.29488					
24	108->128	0.24519	8.0788	153.47	0.0011	-4.4311	-5.9638
	115->128	-0.23367					
	124->128	0.29169					
25	126->144	-0.24473	8.1091	152.90	0.0035	-5.6561	-5.3496
	127->133	0.27532					
	127->136	-0.25012					
	127->138	-0.2477					
26	126->144	0.23958	8.1503	152.12	0.0188	6.1992	4.8957
27	119->129	0.29781	8.2234	150.77	0.0599	7.4504	9.0092
	122->128	-0.23788					
28	115->128	0.2283	8.2456	150.36	0.0150	-11.6791	-12.4143

	119->129	0.24809					
	122->128	0.2656					
29	126->130	0.60733	8.3387	148.69	0.0007	-2.4409	-2.0831
30	127->137	0.2894	8.3566	148.37	0.0020	-0.1342	0.3439
31	120->128	0.39496	8.3823	147.91	0.0010	-2.9283	-3.3651
	121->128	-0.38553					
	122->128	-0.34298					
32	116->130	0.30631	8.3917	147.75	0.0386	49.0154	36.7
	123->130	0.28596					
	125->130	0.32584					
33	127->141	-0.25231	8.4020	147.56	0.0020	-23.9826	-15.8011
	127->143	0.22493					
	127->145	0.26577					
	127->146	0.35144					
34	115->128	0.2926	8.4354	146.98	0.0031	-10.6424	-9.8788
	117->128	-0.22476					
	122->128	-0.22466					
35	126->132	0.2311	8.4466	146.79	0.0111	12.3078	12.9916
	126->134	0.23143					
36	124->129	-0.22815	8.4608	146.54	0.0081	14.2638	13.5725
	125->129	0.28794					

[a]Number of the excited states; [b]Only transitions with contribution over 10.0% were listed; [c]Configuration-interaction coefficient; [d]Excitation energy; [e]Wavelength; [f]Oscillator strength; [g]Rotatory strength in velocity form (10^{-40} cgs); [h]Rotatory strength in length form (10^{-40} cgs).

Table S16. Key transitions, oscillator strengths, and rotatory strengths in the ECD spectrum of conformer 8'R-1-3 at the CAM-B3LYP/6-311G(d) level of theory in MeOH with IEFPCM solvent model.

Num[a]	*Transition*[b]	*CI-coeff*[c]	*ΔE (eV)*[d]	*λ (nm)*[e]	*f*[f]	R_{vel}[g]	R_{len}[h]
1	127->128	0.65529	4.4593	278.04	0.1554	11.2889	11.2721
2	126->128	0.62936	4.9925	248.34	0.2769	-6.2949	-0.8608
	127->129	0.22072					
3	121->128	0.47868	5.5407	223.77	0.0014	0.5692	1.0779
	123->128	0.37119					
4	126->128	-0.25295	5.8867	210.62	0.3432	-6.562	-7.0042
	126->129	0.24967					
	127->129	0.58401					
5	122->130	0.58839	5.9700	207.68	0.0013	-3.3054	-3.3899
	125->130	0.20315					
6	126->129	0.60742	6.2465	198.49	0.6247	28.67	30.274
	127->129	-0.28062					
7	119->128	0.45951	6.6324	186.94	0.0840	26.9978	27.2694
	120->128	0.23517					
	124->128	-0.24885					
8	118->128	-0.23939	6.8632	180.65	0.0892	-6.3924	-8.0035
	119->128	0.4339					
	124->128	0.29846					
9	112->128	-0.19727	7.0901	174.87	0.0519	-12.7443	-15.524
	117->128	0.31633					
	118->128	0.40133					
10	127->131	0.47134	7.3237	169.29	0.0360	2.8509	1.3364
	127->132	-0.26418					

11	124->128	-0.25583	7.3814	167.97	0.0095	-6.7583	-7.3878
	125->128	0.35577					
	127->131	0.23935					
12	112->128	0.40608	7.5013	165.28	0.0121	-5.0463	-6.1503
	118->128	0.19876					
	124->128	0.24618					
13	127->131	0.22667	7.5438	164.35	0.0488	-10.3223	-9.3201
	127->134	0.24371					
	127->135	0.49731					
14	125->130	0.52612	7.6328	162.44	0.0380	20.6458	26.1641
	125->132	-0.21877					
15	108->128	-0.2266	7.6739	161.57	0.0067	-10.1029	-10.1359
	109->128	0.20963					
	121->129	-0.30585					
	123->129	-0.24162					
	125->128	0.3865					
16	109->128	0.25979	7.7772	159.42	0.0139	2.9338	3.7583
	121->129	0.34421					
	124->129	0.24081					
	125->128	0.19609					
17	126->131	0.36659	7.8370	158.20	0.0747	-2.8208	-3.8702
	126->132	-0.21324					
	126->134	-0.2101					
	126->135	-0.24508					
18	108->128	0.20615	7.9165	156.61	0.0052	-14.4139	-11.9953
	123->128	0.28011					
	125->128	0.20213					
	127->130	-0.27838					
	127->133	0.19523					
19	123->128	0.18777	7.9422	156.11	0.0334	-36.006	-37.8589
	127->130	0.48147					
20	120->129	0.22246	7.9496	155.96	0.0623	-15.5794	-15.2179
	123->129	0.35042					
	124->129	-0.25337					
21	125->130	0.21728	7.9792	155.38	0.0174	7.7215	-0.6379
	125->131	0.25375					
	125->132	0.31174					
22	127->130	0.31704	7.9957	155.06	0.0612	-11.9529	-13.0982
	127->133	0.33729					
	127->136	0.32604					
23	126->135	-0.30454	8.0445	154.12	0.0186	15.768	15.7963
	127->130	-0.1956					
	127->134	0.31016					
	127->136	0.24598					
	127->138	-0.22717					
24	108->128	-0.28058	8.0896	153.26	0.0012	-10.6221	-11.1682
	116->128	0.38011					
	120->128	-0.21984					
	124->128	-0.22508					
25	126->131	0.2646	8.1152	152.78	0.0212	6.2243	7.5244
	126->135	0.23679					

	126->144	-0.18941					
	127->134	0.30069					
26	126->131	0.22794	8.1359	152.39	0.0205	7.3157	5.9198
	126->133	-0.25449					
	126->134	0.25975					
	126->144	0.26083					
	126->145	-0.1978					
27	117->129	0.24142	8.2406	150.46	0.0798	26.293	27.3569
	118->129	0.21597					
	119->129	0.47635					
28	115->128	0.22709	8.2566	150.16	0.0015	-3.1968	-3.6484
	120->128	0.37481					
	122->128	-0.32907					
	124->128	0.19736					
29	115->128	0.25673	8.3697	148.13	0.0011	0.0349	0.5127
	122->128	0.44821					
	123->128	-0.24267					
30	126->130	0.56667	8.3951	147.69	0.0039	0.822	0.4862
31	126->130	0.20459	8.4175	147.29	0.0011	1.0707	-5.6525
	127->137	0.35444					
	127->140	-0.19654					
	127->144	-0.23478					
32	120->130	-0.19345	8.4344	147.00	0.0132	39.6955	45.6133
	124->129	0.19715					
	125->129	-0.18888					
33	120->130	-0.19376	8.4418	146.87	0.0078	-22.5728	-23.9355
	126->130	0.23645					
34	127->145	0.40402	8.4839	146.14	0.0015	2.1554	6.8735
	127->147	0.22938					
	127->148	-0.21958					
35	117->129	0.23085	8.5261	145.42	0.0035	-8.3861	-9.0666
	118->129	0.31038					
	119->129	-0.28194					
	124->129	-0.19792					
36	122->128	0.2054	8.5633	144.79	0.0043	7.4281	8.1925
	126->134	0.25419					

[a]Number of the excited states; [b]Only transitions with contribution over 10.0% were listed; [c]Configuration-interaction coefficient; [d]Excitation energy; [e]Wavelength; [f]Oscillator strength; [g]Rotatory strength in velocity form (10^{-40} cgs); [h]Rotatory strength in length form (10^{-40} cgs).

Table S17. Key transitions, oscillator strengths, and rotatory strengths in the ECD spectrum of conformer 8'R-1-4 at the CAM-B3LYP/6-311G(d) level of theory in MeOH with IEFPCM solvent model.

Num[a]	***Transition***[b]	***CI-coeff***[b]	***ΔE (eV)***[d]	***λ (nm)***[e]	***f***[f]	R_{vel}[g]	R_{len}[h]
1	127->128	0.65495	4.4545	278.34	0.1529	-6.6824	-7.5791
2	126->128	0.63262	4.9828	248.82	0.2741	42.1056	43.9927
3	121->128	0.42118	5.5252	224.40	0.0008	0.9367	1.1947
	123->128	0.44891					
4	126->128	-0.24684	5.8848	210.69	0.3259	-13.0944	-14.1663
	126->129	0.25263					
	127->129	0.58579					
5	122->130	0.58725	5.9705	207.66	0.0013	-3.7726	-3.6627

6	126->129	0.60551	6.2326	198.93	0.6201	40.3095	44.7751
	127->129	-0.28604					
7	119->128	0.48131	6.6801	185.60	0.0927	20.0205	19.5446
	121->128	-0.24903					
8	113->128	0.22765	6.8620	180.68	0.0591	-5.7551	-7.3398
	119->128	0.35105					
	124->128	0.30618					
9	118->128	0.5625	7.1173	174.20	0.0629	-19.7742	-10.7755
10	127->131	0.5913	7.3159	169.47	0.0043	11.8734	12.0108
	127->132	-0.29058					
11	125->128	0.38795	7.3418	168.87	0.0123	-9.0927	-8.9452
12	113->128	0.39658	7.4146	167.22	0.0090	4.3207	3.7791
	124->128	-0.34854					
13	127->135	0.5237	7.4930	165.47	0.0831	-8.3521	-7.3467
14	125->130	0.52589	7.6322	162.45	0.0373	20.5422	26.2376
	125->132	-0.22578					
15	121->129	-0.25483	7.6683	161.68	0.0018	-0.4529	-0.5616
	123->129	-0.29208					
	125->128	0.41161					
16	121->129	0.2776	7.7596	159.78	0.0006	3.6641	2.4042
	123->129	0.2852					
17	126->131	0.34332	7.8519	157.90	0.0132	-10.7047	-7.5141
	127->130	-0.24576					
	127->133	0.25451					
	127->134	-0.24876					
18	126->131	0.3665	7.9135	156.67	0.0792	45.4673	46.8842
	126->135	-0.23257					
	127->130	0.23815					
19	116->128	0.22519	7.9178	156.59	0.0057	11.4914	10.6924
	123->128	0.31056					
	125->128	0.24639					
20	126->135	0.3315	7.9336	156.28	0.1409	-60.7098	-66.0998
	127->130	0.34991					
21	125->132	0.26533	7.9694	155.58	0.0110	6.7718	3.5049
	127->130	0.24962					
22	124->129	-0.24204	7.9739	155.49	0.0237	-17.362	-15.4758
	126->135	0.29205					
23	125->132	-0.24029	7.9940	155.10	0.0378	-32.6739	-36.275
	127->130	0.40963					
24	108->128	0.2546	8.0930	153.20	0.0023	-11.6038	-13.39
	116->128	0.31821					
25	127->133	0.266	8.1077	152.92	0.0030	-0.5492	-0.7554
	127->136	0.33212					
	127->138	0.23211					
26	126->133	-0.22414	8.1511	152.11	0.0201	7.644	6.6784
	126->136	-0.24329					
	126->144	0.25398					
27	118->129	0.26239	8.2320	150.61	0.0712	0.9052	2.9686
	119->129	0.43087					
28	120->128	0.38147	8.2658	150.00	0.0017	-4.7414	-5.4261
	122->128	-0.32896					

29	126->130	0.37254	8.3352	148.75	0.0008	-0.8107	-0.4561
	127->137	-0.28811					
30	126->130	0.41928	8.3805	147.94	0.0038	0.5144	-1.1718
	127->137	0.25362					
31	115->128	0.2261	8.3839	147.88	0.0007	1.8153	2.2541
	122->128	0.44332					
	126->130	-0.23369					
32	127->141	-0.23458	8.3981	147.63	0.0017	4.7288	6.4209
	127->143	-0.24996					
	127->146	0.42639					
33	120->130	0.30201	8.4332	147.02	0.0285	28.6011	30.5629
	122->130	-0.25836					
	124->130	0.23645					
	125->130	0.25562					
34	121->129	-0.23673	8.4586	146.58	0.0077	-11.9981	-11.519
	124->129	0.32194					
	125->129	-0.30364					
35	126->130	0.31867	8.4708	146.37	0.0033	6.6517	6.5007
	126->134	-0.24006					
36	118->129	0.47383	8.5579	144.88	0.0014	-7.8999	-8.5001
	119->129	-0.3287					

[a]Number of the excited states; [b]Only transitions with contribution over 10.0% were listed; [c]Configuration-interaction coefficient; [d]Excitation energy; [e]Wavelength; [f]Oscillator strength; [g]Rotatory strength in velocity form (10^{-40} cgs); [h]Rotatory strength in length form (10^{-40} cgs).
